# Cost-Utility Analysis of Accelerated and Standard Strategies for Renal Replacement Therapy Initiation

**DOI:** 10.1001/jamanetworkopen.2025.35343

**Published:** 2025-10-03

**Authors:** Jeff Round, Ilke Akpinar, Charles Yan, Natasha Patel, Sasha van Katwyk, Carmel Montgomery, Ron Wald, Sean M. Bagshaw

**Affiliations:** 1 Department of Pediatrics, Faculty of Medicine and Dentistry, University of Alberta, Edmonton, Alberta, Canada; 2 Faculty of Nursing, University of Alberta, Edmonton, Alberta, Canada; 3 Institute of Health Economics, Edmonton, Alberta, Canada; 4 Division of Nephrology, St Michael’s Hospital, Toronto, Ontario, Canada; 5 Li Ka Shing Knowledge Institute of St Michael’s Hospital, Toronto, Ontario, Canada; 6 Department of Nephrology and Hypertension, Tel Aviv Medical Center, Tel Aviv, Israel; 7 Department of Critical Care Medicine, Faculty of Medicine and Dentistry, University of Alberta and Alberta Health Services, Edmonton, Alberta, Canada

## Abstract

**Importance:**

Little is known about the long-term costs and outcomes related to strategies for timing of initiation of kidney replacement therapy (KRT) in critically ill patients with severe acute kidney injury (AKI).

**Objective:**

To estimate the cost-utility and cost-effectiveness of accelerated KRT initiation compared with standard KRT initiation in critically ill patients with AKI.

**Design, Setting, and Participants:**

In this economic evaluation, a state-transition model was developed using data from the Standard vs Accelerated Initiation of Renal Replacement Therapy in AKI (STARRT-AKI) trial, a multicenter, multinational randomized clinical trial of critically ill patients with severe AKI conducted between October 2015 and September 2019. Trial data were linked to administrative health databases in Alberta, Canada, to estimate costs and long-term clinical outcomes. The model included 4 health states: no chronic kidney disease, severe chronic kidney disease, KRT dependent, and dead. Costs are reported in 2024 Canadian dollars. Data were analyzed from February 2022 to November 2024.

**Exposure:**

Initiation of KRT.

**Main Outcomes and Measures:**

The primary outcome for the economic evaluation was cost per quality-adjusted life-year (QALY) gained. The QALY is a combined measure of patient quality of life and length of life. Expected costs, QALYs, incremental cost-effectiveness ratio (ICER), and incremental net monetary benefit (INMB) were estimated on the basis of 5000 Monte Carlo simulations.

**Results:**

A total of 146 patients from the STARRT-AKI trial were included in the analysis, with 73 patients (mean [SD] age, 59.67 [14.5] years; 52 men [71.3%]) randomized to receive accelerated initiation and 73 patients (mean [SD] age, 61.88 [12.9] years; 48 men [65.8%]) randomized to receive standard initiation. Standard initiation was more costly per patient than accelerated initiation (mean [SD], $251 370 [$155 801] vs $231 518 [$183 302]) but generated more QALYs (mean [SD] 7.49 [2.03] QALYs vs 6.64 [1.76] QALYs). The ICER of standard initiation compared with accelerated initiation was $23 208, with an INMB of $22 648 (95% credible interval, $15 980-$29 316) when assuming a willingness to pay per QALY of $50 000.

**Conclusions and Relevance:**

The findings of this economic evaluation suggest that standard KRT initiation may be cost-effective in a Canadian setting, but this finding was sensitive to postdischarge cost trajectories and regional variation in KRT dependence.

## Key Points

**Question:**

Is accelerated initiation of kidney replacement therapy cost-effective compared with standard initiation in critically ill patients with acute kidney injury?

**Findings:**

This economic evaluation of 146 patients found that standard initiation of kidney replacement therapy is cost-effective compared with accelerated initiation, with an incremental cost-effectiveness ratio of $22 648 per quality-adjusted life-year (costs measured in 2024 Canadian dollars).

**Meaning:**

These findings suggest that standard initiation of kidney replacement therapy is a cost-effective use of health care resources when compared with accelerated initiation.

## Teaser

This economic evaluation examines the cost-utility and cost-effectiveness of accelerated compared with standard renal replacement therapy (RRT) initiation in critically ill patients with acute kidney injury.

## Introduction

Acute kidney injury (AKI) is a common and serious clinical condition characterized by a rapid decline in kidney function leading to the accumulation of metabolic waste, electrolyte imbalances, and volume dysregulation.^1^ AKI is often multifactorial and characterized by a spectrum of risk factors in critically ill patients. AKI has an incidence of more than 50% in critically ill patients,^2^ is associated with substantial morbidity and mortality,^3^ and recent data suggest that its incidence is increasing.^3,4^ When AKI is complicated by medically refractory metabolic, uremic, or fluid derangements, kidney replacement therapy (KRT) is often initiated.^5^ However, when AKI is not accompanied by these complications (ie, urgent indications), the benefit of early or accelerated KRT is not clear, and the most effective strategy for when to start KRT has remained uncertain.^6,7^

The Standard vs Accelerated Initiation of Renal Replacement Therapy in AKI (STARRT-AKI) study was a multinational, open-label, randomized clinical trial (RCT)^5^ that compared 2 strategies for KRT initiation in critically ill patients with severe AKI: an accelerated strategy that entailed commencing KRT in the absence of a conventional indication vs a standard strategy that constituted careful observation and KRT initiation if a conventional indication developed or if AKI was unremitting and the clinician perceived starting KRT was in the patient’s best interest. STARRT-AKI found that accelerated KRT initiation did not confer a lower risk of death at 90 days compared with standard initiation (relative risk, 1.00; 95% CI, 0.93-1.09).^5^ However, there were important differences in the key secondary outcomes, in particular, a greater risk of KRT dependence at day 90 for those allocated to the accelerated compared with standard strategy (relative risk, 1.74; 95% CI, 1.24-2.43).^5^

Acute KRT is invasive, resource intensive, and expensive. Patients receiving acute KRT generally have longer stays in the intensive care unit (ICU) and hospital compared with those not treated with KRT.^4^ The decision to commence KRT may have downstream implications on the longer term risk for chronic kidney disease (CKD), kidney failure (eg, requiring maintenance dialysis), and overall health status,^5^ all of which can influence health system resource use and costs. Accordingly, understanding both the short-term and long-term clinical outcomes and health economic implications are important. Here, we report a cost-utility analysis comparing accelerated and standard strategies for KRT initiation using data from STARRT-AKI trial participants in Alberta, Canada.

## Methods

### Study Population, Setting, and Location

STARRT-AKI was a multinational, open-label, RCT of 3019 critically ill patients with severe AKI conducted between October 2015 and September 2019 at 168 hospitals in 15 countries, comparing accelerated and standard approaches to KRT initiation on the risk of death and dialysis dependence at 90 days. The study protocol, statistical analysis plan, and main trial findings are described elsewhere.^5,8,9^ This economic evaluation included participants at 9 sites in Alberta, Canada. STARRT-AKI was approved by the health research ethics boards at the University of Alberta, Unity Health Toronto, and research ethics boards at all participating sites. All participants (or legally authorized representatives) provided written consent to participate in the RCT and use of long-term administrative data up to 1 year after the trial for this purpose.

### Model Structure and Cost-Utility Analysis

This economic evaluation followed International Society for Pharmacoeconomics and Outcomes Research (ISPOR) Good Research Practices Task Force report for cost-effectiveness analysis alongside clinical trials.^10,11^ We developed a decision analytic Markov model to simulate lifetime costs and quality-adjusted life-years (QALYs) for each trial group. Data from the STARRT-AKI trial were used to estimate patient costs, outcomes, and model transition probabilities.

The Markov model consists of 4 discrete health states defined by kidney function (estimated glomerular filtration rate [eGFR]), receipt of KRT, and death, all at 90 days: (1) no CKD (eGFR ≥60 mL/min/1.73 m^2^), (2) CKD and not KRT dependent (non–dialysis dependent with eGFR <60 mL/min/1.73 m^2^), (3) alive and KRT dependent, and (4) dead. The model starts 90 days after randomization, which is defined as the post-AKI period. This time point was selected to align with the timing of the trial primary outcome, and costs and outcomes from the period between randomization to day 90 are included in the analysis. A patient will be in 1 of the no CKD, CKD and not KRT dependent, or alive and KRT dependent health states for the initial model cycle. They can be in only 1 of these health states at any given time, but over their lifetime the patients can either stay in the same health state or transition to another health state, including dead. A patient could transition to dead at any point. The model is run independently for participants allocated to the accelerated and standard strategy groups, respectively. The Markov model structure showing the possible transitions between health states is presented in the Figure. Data extracted from the trial were linked with administrative databases and were applied as analytic inputs for the decision model. A cycle length of 1 month (12 cycles per year, approximately 30 days per cycle) was determined to be the shortest clinically relevant period. A lifetime horizon for estimating costs and benefits was adopted, and the model was run for 480 cycles (40 years) to capture lifetime costs and QALYs. The Markov trace figures presented in eFigure 1 in Supplement 1 show that it was sufficient for all simulated patients to have entered the dead state. The starting age for patients in the model was 61 years based on the average age of the trial population. Cost-utility analysis results are presented as incremental costs, incremental QALYs, incremental cost effectiveness ratios (ICERs), and incremental net monetary benefits (INMBs).

**Figure.**
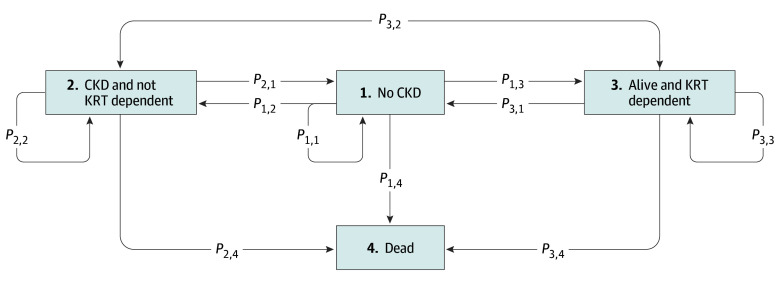
Markov Model Structure The figure shows the possible transitions between health states. CKD indicates chronic kidney disease; KRT, kidney replacement therapy; *P*, probability.

### Measurement and Valuation of Resources and Costs

We estimated resource use using 7 linked health care administrative databases in Alberta: The National Ambulatory Care Reporting System,^12^ Discharge Abstract Database,^13^ Practitioner Claims,^14^ Pharmaceutical Information Network,^15^ Laboratory Testing Database,^14^ and Population Registry and Vital Statistics.^14^ A full list of administrative data elements used in the analysis is provided in the eAppendix and eTables 1 to 10 in Supplement 1.

Costs were estimated from a Canadian health system perspective using national estimates of the costs of activities, supplemented by cost data from Alberta where necessary. Health care costs were defined as the sum of ambulatory care, hospitalization, physician claims, and drug costs. We obtained drug unit prices from the Alberta Drug Benefit List^15^ and multiplied these by the units of medication dispensed to estimate drug costs. Hospital costs were calculated by multiplying the resource intensity weight for each hospital stay or emergency department or outpatient visit by the Canadian Institute for Health Information cost of a standard hospital stay in 2022,^16^ the most recent year for which data were available ($9220). The resource intensity weight is used to adjust individual patient costs to account for the intensity and complexity of care needed for individual encounters.^17^

Estimates of the cost per cycle that included an acute ICU admission were significantly higher compared with only non-ICU costs, a function of the high costs of care in ICU irrespective of whether KRT is used. Our analysis suggested health care cost stabilized approximately 4 months after KRT initiation. Consequently, our Markov model treated these costs separately. Specifically, we considered onetime costs incurred from the index admission to 90 days. Costs incurred during the fourth month after admission were applied exclusively to that month. Average monthly costs from the fifth month up to 1 year were applied uniformly to the remaining months. Ambulatory care, inpatient, physician, and drug costs for each patient were then summed according to previously defined health-states to have total health care costs. All costs were reported in 2024 Canadian dollars using the Canada Consumer Price Index.^18^ Both costs and utilities were discounted using an annual rate of 1.5%.^19^

### Quality of Life and QALYs

Health-related quality-of-life (HRQL) was measured using the EuroQuol 5-Dimension 5-Level (EQ-5D-5L) questionnaire and was valued using the Canadian tariff.^20^ Patients completed the EQ-5D-5L questionnaires on day 90 and on day 365 after randomization. The EQ-5D-5L measures HRQL across 5 dimensions (mobility, self-care, usual activities, pain and discomfort, and anxiety and depression) with 5 levels of scoring per domain. Using a scoring algorithm, the individual responses to each of the domains were transformed into health utility scores, where 0 represents dead and 1 represents the best health state.^20^

QALYs were calculated for each intervention by weighting the utility scores by time spent in that health state. Because of the small sample size for some of the health states at 365 days (50 of 98 available patients completed the EQ-5D-5L at 365 days), we supplemented HRQL data from the trial with utility values from the literature.^21^ Using a recently published systematic review on HRQL utility weights for economic evaluation through different stages of CKD,^21^ we reviewed the primary studies to identify the most relevant utility values that matched our population of patients with new CKD, where HRQL was measured using the EQ-5D-5L and where the research took place in a similar health system setting. We identified 2 studies that reported EQ-5D-5L utility values for new CKD patients, including 1 Alberta-based study and 1 study from the UK, which also has a publicly funded health system.^22,23^ The utility values used in our analysis also align with recent economic analysis inputs in this disease area.^24^

### Transition Probabilities

Transition probabilities were calculated from the STARRT-AKI trial data and Alberta Health Administrative Databases using monthly KRT status and eGFR value for each patient. To calculate the transition probabilities for the 4-state Markov model, patients were categorized into 1 of the 4 health states at 2 time points according to 90-day (time 1) and 365-day (time 2) eGFR value: no CKD, CKD and not KRT dependent, alive and KRT dependent, and dead. The transition probabilities were organized into a 4 × 4 transition matrix *PP*, where each element *P_ij_P_ij_* represents the probability of transitioning from state *ii* at time 1 to state *jj* at time 2. The transition probability *P_ij_P_ij_* was calculated as the proportion of patients in state *ii* at time 1 who were observed in state *jj* at time 2 and adjusted to a monthly value. For example, *P*_12_*P*_12_ represents the probability of transitioning from no CKD to CKD and not KRT dependent, whereas *P*_14_*P*_14_ represents the probability of transitioning from no CKD to dead. Similarly, *P*_33_*P*_33_ reflects the probability of remaining in the alive and KRT-dependent state.

Patients who were classified as dead at time 1 remained in that state, leading to a terminal state in the model where *P*_44_ = 1. This transition matrix formed the foundation for subsequent long-term projections in the Markov model. For events that were not time dependent, we estimated the probability of the event occurring and fit beta distributions to characterize the sampling uncertainty. For time-dependent probabilities, we conducted 5000 simulations for each cycle for each parameter, resulting in a 480 × 5000 matrix. The mean value was calculated across the 5000 simulations for each cycle, generating a vector of means for the 480 cycles for the probability. From this vector, we calculated the mean and SD to define the sampling distribution for that cycle.

### Statistical Analysis

Data were analyzed from February 2022 to November 2024. Patient characteristics stratified by allocated KRT strategy were summarized using mean (SD), counts, and percentages, as appropriate. Survival time stratified by KRT strategy was estimated using a Kaplan-Meier curve and was compared between patients in the accelerated and standard groups using a log-rank test. Probabilistic sensitivity analysis was used to reflect sampling and measurement uncertainty in the underlying model data. We fit γ distributions to cost data and beta distributions to transition probabilities and utilities to estimate the average costs and health outcomes for each strategy. Expected costs, QALYs, ICER, and INMB were estimated on the basis of 5000 Monte Carlo simulations. The INMB values were calculated using a willingness-to-pay (WTP) threshold of $50 000 per QALY, based on a commonly referenced threshold used in Canada.^25^ For model-based results, we report 95% bayesian credible intervals (CrI); for all other analyses, we report 95% CIs or SD.

Additional 1-way sensitivity analyses were performed to examine the impact of varying (1) the percentage of patients who were KRT dependent at 90 days and (2) the monthly costs of treatment. In the base case analysis, patients were allocated to the KRT-dependent model state according to data from Alberta study participants, where 2.1% of patients in the accelerated initiation group and 9.8% in the standard initiation group were receiving KRT at day 90. An alternative scenario used data from a broader population within the trial, reporting that KRT dependence among survivors at 90 days (56%) was 10.4% for accelerated initiation and 6.0% for standard initiation.^5^ The second factor examined was the impact of monthly costs. An alternative scenario assumed equal monthly costs for both groups beyond the third month. All analyses were performed using Stata statistical software version 14 (StataCorp) and R statistical software version 4.2.2 (R Project for Statistical Computing). Between-group differences were tested using a 2-sample *t* test using R function tsum.test for continuous variables and by Pearson χ^2^ test using R function chisq.test for categorical variables at a significance level of *P* < .05.

## Results

Specific cost values for the no CKD, CKD and not KRT dependent, and KRT states are given in Table 1. Utility scores and transition probabilities by health state are shown in Table 2. One hundred forty-six patients were included in the analysis, with 73 patients (mean [SD] age, 59.67 [14.5] years; 52 men [71.3%]) randomized to receive accelerated initiation and 73 patients (mean [SD] age, 61.88 [12.9] years; 48 men [65.8%]) randomized to receive standard initiation for KRT initiation (Table 3). Of these, 50 patients (68.5%) in the accelerated initiation group and 57 (78.1%) in the standard initiation group survived at day 90 (eTable 1 in Supplement 1). Among the survivors at 90 days, 2.1% (1 patient) of those allocated to accelerated initiation and 9.8% (5 patients) of those allocated to standard initiation were dialysis dependent (eTable 1 in Supplement 1). The mean (SD) costs from index admission to 90 days were $182 626 ($175 710) for accelerated initiation and $161 601 ($104 048) for standard initiation (Table 1). EQ-5D-5L data were available for 43 patients at 90 days, and mean utility scores were 0.763 (95% CI, 0.657-0.869; 20 patients) for accelerated initiation and 0.710 (95% CI, 0.616-0.805; 23 patients) for standard initiation.

**Table 1.** Health State Costs

KRT strategy and period	Cost, mean (SD), $^a^			
	Total^b^	Alive and KRT dependent^c^	CKD, not KRT dependent^c^	No CKD^c^
Accelerated KRT initiation^d^				
0-90 d	182 626 (175 710)	NA	NA	NA
91-120 d	28 486 (126 302)	20 299 (90 000)	4709 (20 880)	3478 (15 423)
>120 d	3293 (4335)	2346 (3089)	544 (717)	402 (529)
Standard KRT initiation^d^				
0-90 d	161 601 (104 048)	NA	NA	NA
91-120 d	9141 (25 820)	6514 (18 398)	1511 (4268)	1116 (3153)
>120 d	5558 (8280)	3960 (5900)	919 (1369)	679 (1011)

Abbreviations: CKD, chronic kidney disease; KRT, kidney replacement therapy; NA, not applicable.

^a^ Health care costs were defined as the sum of ambulatory care, hospitalization, physician services, and drug expenditures. Hospital costs were estimated by multiplying the resource intensity weight by the 2022 average cost of a standard hospital stay ($9220). Physician costs reflected actual payments to physicians. Drug costs were estimated by multiplying the quantity of medication dispensed by the corresponding unit prices. All costs were then adjusted to 2024 Canadian dollars using the Consumer Price Index.

^b^ Cost during the first 90 days, monthly cost during 91 to 120 days, and monthly cost during 121 days to 1 year after the index admission are shown.

^c^ Costs for alive and KRT dependent, CKD and not KRT dependent, and no CKD states are calculated by multiplying the total cost by the respective ratios of each state to the total cost, estimated from Alberta data. Alive and KRT dependent accounted for 71% of the total monthly cost, CKD and not KRT dependent accounted for 17% of the total, and no CKD accounted for 12% of the total.

^d^ Zero to 90 days refers to index admission to day 90, 91 to 120 days refers to the first month after day 90, and greater than 121 days refers to day 120 up to 1 year after the index admission.

**Table 2.** Utility Scores and Transition Probabilities

Utility values	Utility value, mean (α, β)^a^	Distribution
No CKD^b^	0.85 (80.00, 14.40)	Beta
CKD and not KRT dependent^b^	0.74 (49.90, 18.00)	Beta
Alive and KRT dependent^c^	0.60 (44.80, 30.00)	Beta
Transition probabilities^d^		
No CKD to alive and KRT dependent	0.001 (0.059, 56.413)	Beta
CKD and not KRT dependent to no CKD (standard)	0.139 (0.396, 2.479)	Beta
CKD and not KRT dependent to no CKD (accelerated)	0.063 (0.110, 1.659)	Beta
CKD and not KRT dependent to alive and KRT dependent	0.016 (0.064, 4.265)	Beta
Alive and KRT dependent to no CKD	0.073 (0.359, 4.379)	Beta
Alive and KRT dependent to CKD and not KRT dependent	0.063 (0.036, 0.521)	Beta
Time-dependent transition probabilities, mean (range)^d^		
No CKD to CKD and not KRT dependent	0.001 (0.000-0.017)	NA
No CKD to dead	0.022 (0.001-0.990)	NA
CKD and not KRT dependent to dead	0.030 (0.003-0.990)	NA
Alive and KRT dependent to dead	0.033 (0.005-0.990)	NA

Abbreviations: AKI, acute kidney injury; CKD, chronic kidney disease; KRT, kidney replacement therapy; NA, not applicable.

^a^ The α and β are the shape parameters of a beta distribution.

^b^ Values were obtained from Jesky et al.^22^

^c^ Values were obtained from Manns et al.^23^

^d^ Calculated according to the data of 146 patients enrolled in the Standard vs Accelerated Initiation of Renal Replacement Therapy in AKI trial in Alberta, Canada.^5^

**Table 3.** Patient Characteristics at Baseline by Study Group

Characteristic	Patients, No. (%)		*P* value^a^
	Accelerated initiation (n = 73)	Standard initiation (n = 73)	
Age, mean (SD), y	59.67 (14.5)	61.88 (12.9)	.33
Sex			
Female	21 (28.7)	25 (34.2)	.59
Male	52 (71.3)	48 (65.8)	
Preexisting conditions			
Hypertension	40 (54.7)	46 (63.0)	.40
Diabetes	23 (31.5)	22 (30.1)	>.99
Heart failure	11 (15.0)	17 (23.9)	.29
Coronary artery disease	13 (17.8)	17 (23.3)	.54
Liver disease	9 (12.3)	9 (12.3)	>.99
Metastatic cancer	0	0	>.99
Admission category			
Scheduled surgery	4 (5.5)	6 (8.2)	.75
Unscheduled surgery	17 (23.3)	10 (13.7)	.20
Medical	52 (71.2)	57 (78.1)	.45
Hospital details diagnostic category			
Cardiovascular	13 (17.8)	10 (13.7)	.65
Gastrointestinal or hepatic	19 (26.3)	12 (16.4)	.22
Metabolic	2 (2.7)	2 (2.7)	>.99
Neurologic	3 (4.1)	5 (6.9)	.72
Respiratory	14 (19.2)	25 (34.2)	.06
Sepsis	20 (27.4)	17 (23.3)	.70
Trauma	0	1 (1.4)	>.99
Other, specify	2 (2.7)	1 (1.4)	>.99
Hospital-acquired risk factor for acute kidney injury in the previous week			
Cardiopulmonary bypass	2 (2.7)	1 (1.4)	>.99
Aortic surgery	2 (2.7)	3 (4.1)	>.99
Other vascular surgery	0	0	>.99
Major trauma	2 (2.7)	2 (2.7)	>.99
Exposure to radiocontrast material	12 (16.4)	7 (9.6)	>.99
Clinical condition at randomization			
Sequential Organ Failure Assessment score, mean (SD)	12.96 (2.9)	13.06 (3.2)	.70
Mechanical ventilation	63 (86.3)	64 (87.7)	>.99
Vasoactive support, mean (SD), d	10.84 (6.9)	10.19 (6.3)	.55
Serum creatinine, mean (SD), mg/dL	3.53 (1.4)	3.49 (1.2)	.85

SI conversion factor: To convert creatinine to micromoles per liter, multiply by 88.4.

^a^ Between-group differences are tested using a 2-sample *t* test using R statistical software function tsum.test for continuous variables, and Pearson χ^2^ test using R function chisq.test for categorical variables at a 5% significance level.

Over a lifetime time horizon, the base-case analysis indicated that standard initiation was more costly per patient than accelerated initiation (mean [SD], $251 370 [$155 801] vs $231 518 [$183 302]; difference, $19 852) (Table 4). Standard initiation also generated more QALYs compared with accelerated initiation (mean [SD], 7.49 [2.03] QALYs vs 6.64 [1.76] QALYs; difference, 0.86 QALYs). These differences translate to an ICER of $23 208 per QALY, comfortably below the commonly used WTP threshold of $50 000 per QALY.^25^ Standard initiation had an estimated INMB of $22 648 (95% CrI, $15 980-$29 316) when assuming the same WTP threshold. Standard initiation was more likely to be cost-effective when compared to accelerated initiation for WTP per QALY values above approximately $34 000.

**Table 4.** Cost-Utility Analysis Results

Analysis and group	Cost, mean (SD), $^a^	Outcome, mean (SD), QALYs	Incremental cost, mean (SD), $^a^	Incremental QALYs, mean (SD)	ICER, $/QALY^a^	IMNB, mean (95% CrI), $^a^
Base case analysis						
Accelerated KRT initiation	231 518 (183 302)	6.64 (1.76)	NA	NA	NA	NA
Standard KRT initiation	251 370 (155 801)	7.49 (2.03)	19 852 (242 167)	0.86 (1.88)	23 208	22 648 (15 980 to 29 316)
Sensitivity analysis, varying percentage in KRT state at day 90						
Accelerated KRT initiation	233 321 (183 330)	6.48 (1.75)	NA	NA	NA	NA
Standard KRT initiation	249 597 (155 792)	7.62 (2.03)	16 276 (242 202)	1.15 (1.88)	14 205	40 724 (34 055 to 47 393)
Sensitivity analysis, varying monthly costs						
Accelerated KRT initiation	231 815 (183 291)	6.61 (1.75)	NA	NA	NA	NA
Standard KRT initiation	215 774 (155 066)	7.38 (2.02)	−16 041 (237 113)	0.77 (1.89)	Dominant	54 541 (47 975 to 61 107)

Abbreviations: CrI, credible interval; ICER, incremental cost-effectiveness ratio; IMNB, incremental monetary net benefit; KRT, kidney replacement therapy; NA, not applicable; QALY, quality-adjusted life-year.

^a^ Costs are given in 2024 Canadian dollars.

### One-Way Sensitivity Analysis

In the 1-way sensitivity analysis based on the total STARRT-AKI trial population for the outcome of 90-day KRT dependence, standard initiation remained more costly and yielded more QALYs than the accelerated strategy, with an ICER of $14 205 and INMB of $40 724 (95% CrI, $34 055-$47 393) at a WTP of $50 000 per QALY (Table 4). In this scenario, standard initiation is most likely to be cost-effective when compared with accelerated strategy at WTP values greater than $22 500 (eFigure 2 in Supplement 1). In the sensitivity analysis assuming equal monthly costs beyond the third month (90 days), standard initiation was less costly than accelerated initiation and produced more QALYs (Table 4). In this scenario, standard strategy was more likely to be cost-effective relative to accelerated initiation at all levels of WTP per QALY thresholds tested (eFigure 3 in Supplement 1).

## Discussion

In this economic evaluation, incremental cost-utility analysis showed that standard initiation of KRT in critically ill patients with severe AKI was cost-effective compared with accelerated initiation. Over a lifetime time horizon, standard KRT initiation would be estimated to cost, on average, $19 852 more than accelerated KRT initiation; however, standard initiation would also generate an estimated 0.86 more QALYs, implying that standard initiation is incrementally cost-effective (ICER, $23 208). However, we also found that accelerated initiation was more likely to be cost-effective at lower values of WTP per QALY, whereas standard initiation was more likely to be cost-effective once the WTP per QALY exceeds $34 000.

A key driver of our findings is the distribution of patients across starting health states in our Markov model. As expected, there was variation in the distribution of these health states across sites and health jurisdictions participating in the trial.^26^ For example, among patients alive at 90 days who were enrolled in participating sites in Alberta, KRT dependence was 2.1% in those allocated to accelerated initiation and 9.8% in those allocated to the standard strategy. This differs both from all Canadian trial sites (KRT dependence of 13.5% of patients in accelerated initiation and 6.4% in standard initiation) and the global trial cohort (10.4% accelerated initiation, 6.0% standard initiation).^5^

Although we have used the 90-day outcome data of the Alberta population for our base-case analysis to align with the available patient-level data on resource use and costs, these outcomes do not align with the overall observations in the STARRT-AKI trial.^26^ To explore the impacts of this variation on 90-day outcomes across jurisdictions, we performed a sensitivity analysis where we allocated patients to starting states in the Markov model on the basis of the main STARRT-AKI trial results. The aim of this sensitivity analysis was to better understand the effect of model starting state on expected lifetime costs and utilities for each KRT initiation strategy in the trial and largely aligned with our primary analysis, showing that standard initiation was more costly but also more cost-effective than the accelerated strategy.

We recommend that our findings be interpreted with regard for local context. Although the use of the overall trial data to determine starting states may be more accurate than the use of the smaller cohort from Alberta alone, we did not have individual patient–level resource use and costing data from participating jurisdictions other than Alberta. It cannot be assumed that Alberta resource use and cost values are representative of costs from other jurisdictions. Given the large number of countries involved in the study, it is difficult to estimate a single global estimate of cost-effectiveness owing to wide variation in health care system structure, costs and payment models.

### Strengths and Limitations

Our economic evaluation has notable strengths. First, our analysis is based on a rigorous, multinational, RCT. Second, we used high-quality data captured in parallel with the STARRT-AKI trial in our Markov model, which reduced the number of assumptions required about data and model structure. Finally, we followed standard health economic evaluation reporting guidelines to strengthen this study’s reporting quality, providing accurate information to guide interpretation for policy and decision-makers.^10^

This study also has limitations that should be mentioned. Our economic analysis is derived from resource use and outcome data from a small sample (5.0%) of all patients enrolled in the STARRT-AKI trial. This reduced the number of observations in total, in particular, the number of patients with data from day 365. The quality-of-life data collected at 90 days and 365 days during the clinical trial were also limited, owing to the high mortality observed (aggregate 90-day mortality was 43.8%).^5^ As such, the calculation of utility scores at day 365 was less reliable, especially for patients in the CKD and not KRT dependent CKD and alive and KRT dependent health states, respectively. To compensate, we leveraged existing utility values for the economic evaluation through different stages of CKD from a recently published systematic review on HRQL utility weights.^21^ We reviewed primary studies included in the systematic review to identify the most relevant utility values according to population, setting and HRQL instrument used to measure outcomes.^22,23^ The utility values used in our analysis also align with recent economic analysis inputs in this disease area.^24^

An additional challenge arising from the smaller sample size of our population was that the clinical outcomes for the population included in the economic evaluation differed from the overall study outcomes. We address this through the reported sensitivity analysis and note that if the Alberta population outcomes were the same as the trial-wide outcomes, standard initiation would have a lower ICER and greater INMB, suggesting a greater likelihood of cost-effectiveness.

## Conclusions

The findings of this economic evaluation suggest that standard KRT initiation among critically ill patients with severe AKI and no urgent indications may be cost-effective in a Canadian setting compared with accelerated KRT initiation. However, our findings were sensitive to assumptions about postdischarge cost trajectories and will likely vary with regional variation in KRT dependence. The findings of this economic evaluation can inform health policy, health system planning, and decision-making related to acute KRT, although inferences beyond a Canadian context should be made with caution.

## Notes

Supplement 1. eTable 1. Patient renal replacement therapy dependence and survival status by arm (total and percentage) eFigure 1. Cost-effectiveness acceptability curve, base case analysis eFigure 2. Cost-effectiveness acceptability curve, sensitivity analysis, varying % in RRT state at 90 days eFigure 3. Cost-effectiveness acceptability curve, sensitivity analysis, varying monthly costs eAppendix. Data elements eTable 2. Ambulatory Care (NACRS) eTable 3. Inpatient (DADS) eTable 4. Long term care eTable 5. Pharmaceutical Information Network (PIN) eTable 6. Population registry—cumulative eTable 7. Population registry—monthly eTable 8. Stakeholder registry eTable 9. Practitioner claims (all) eTable 10. Laboratory

Supplement 2. STARRT-AKI Investigators

Supplement 3. Data Sharing Statement
