# Supplementary material for: Cost-Utility Analysis of Accelerated and Standard Strategies for Renal Replacement Therapy Initiation
Source: JAMA Netw Open. 2025 Oct 3;8(10):e2535343. doi: 10.1001/jamanetworkopen.2025.35343 (PMC12495491; doi:10.1001/jamanetworkopen.2025.35343)
Supplement: Supplement 1. — eTable 1. Patient renal replacement therapy dependence and survival status by arm (total and percentage) eFigure 1. Cost-effectiveness acceptability curve, base case analysis eFigure 2. Cost-effectiveness acceptability curve, sensitivity analysis, varying % in RRT state at 90 days eFigure 3. Cost-effectiveness acceptability curve, sensitivity analysis, varying monthly costs eAppendix. Data elements eTable 2. Ambulatory Care (NACRS) eTable 3. Inpatient (DADS) eTable 4. Long term care eTable 5. Pharmaceutical Information Network (PIN) eTable 6. Population registry—cumulative eTable 7. Population registry—monthly eTable 8. Stakeholder registry eTable 9. Practitioner claims (all) eTable 10. Laboratory [file jamanetwopen-e2535343-s001.pdf]

Supplemental Online Content

Round J, Akpinar I, Yan C, et al; STARRT-AKI Investigators. Cost-utility analysis of accelerated and standard strategies for renal replacement therapy initiation. *JAMA Netw Open*. 2025;8(10):e2535343. doi:10.1001/jamanetworkopen.2025.35343

eTable 1. Patient renal replacement therapy dependence and survival status by arm (total and percentage)

eFigure 1. Cost-effectiveness acceptability curve, base case analysis

eFigure 2. Cost-effectiveness Acceptability Curve, sensitivity analysis, varying % in RRT state at 90 days

eFigure 3. Cost-effectiveness acceptability curve, sensitivity analysis, varying monthly costs

eAppendix. Data elements

eTable 2. Ambulatory Care (NACRS)

eTable 3. Inpatient (DADS)

eTable 4. Long term care

eTable 5. Pharmaceutical Information Network (PIN)

eTable 6. Population registry—cumulative

eTable 7. Population registry—monthly

eTable 8. Stakeholder registry

eTable 9. Practitioner claims (all)

eTable 10. Laboratory

This supplemental material has been provided by the authors to give readers additional information about their work.

© 2025 Round J et al. *JAMA Network Open*.

eTable 1: Patient renal replacement therapy dependence and survival status by arm (total and percentage)

	**Accelerated initiation arm**	**Standard initiation arm**
Number of patients	73 (100%)	73 (100%)
Number of patients who died in ICU	23 (31.51%)	16 (21.92%)
Number of patients discharged from ICU	50 (68.49%)	57 (78.08%)
After 7 days of ICU discharge number of RRT-dependent patients	12 (24.00%)	12 (21.05%)
After 7 days of ICU discharge number of RRT independent patients	38 (76.00%)	45 (78.95%)
After ICU discharge RRT independent patients who died before day 90	1 (2.63%)	5 (11.11%)
After ICU discharge RRT independent patients who remained RRT-independent on day 90	37 (97.37%)	39 (86.67%)
After ICU discharge RRT independent patients who required RRT on day 90	0 (0.00%)	1 (2.22%)
After ICU discharge RRT dependent patients who died before day 90	1 (8.33%)	2 (16.67%)
After ICU discharge RRT dependent patients who did not require RRT on day 90	10 (83.33%)	6 (50.00%)
After ICU discharge RRT dependent patients who required RRT on day 90	1 (8.33%)	4 (33.33%)
Number of RRT independent patients on day 90	47 (97.92%)	45 (90.00%)
Number of RRT-dependent patients on day 90	1 (2.08%)	5 (10.00%)
Day 90 RRT independent patients who remaining RRT independent state on day 365	41 (87.23%)	37(82.22%)
Day 90 RRT independent patients who required RRT on day 365	0 (0.00%)	1 (2.22%)

© 2025 Round J et al. *JAMA Network Open*.

Day 90 RRT independent patients not available/ unknown on day 365	3 (6.38%)	7 (15.56%)
Day 90 RRT independent patients not applicable on day 365 (deceased)	3 (6.38%)	0 (0.00%)
Day 90 RRT-dependent patients who did not need RRT on day 365	0 (0.00%)	1 (20.00%)
Day 90 RRT-dependent patients who need RRT on day 365	0 (0.00%)	2 (40.00%)
Day 90 RRT dependent patients not applicable on day 365 (deceased)	1 (100.00%)	2 (40.00%)

© 2025 Round J et al. *JAMA Network Open*.

eFigure 1: Cost-effectiveness acceptability curve, base case analysis

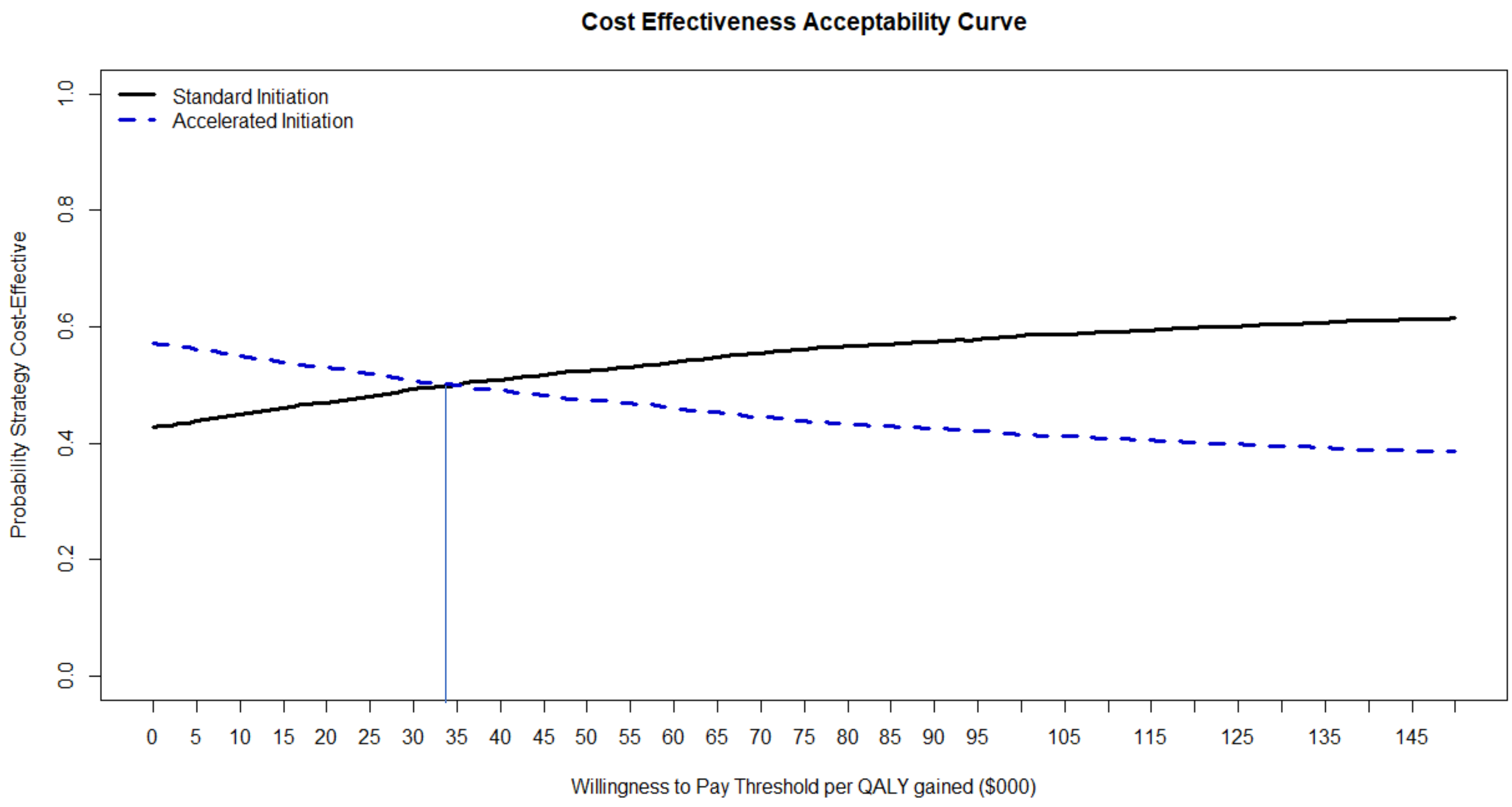

© 2025 Round J et al. *JAMA Network Open*.

eFigure 2: Cost-effectiveness Acceptability Curve, sensitivity analysis, varying % in RRT state at 90 days

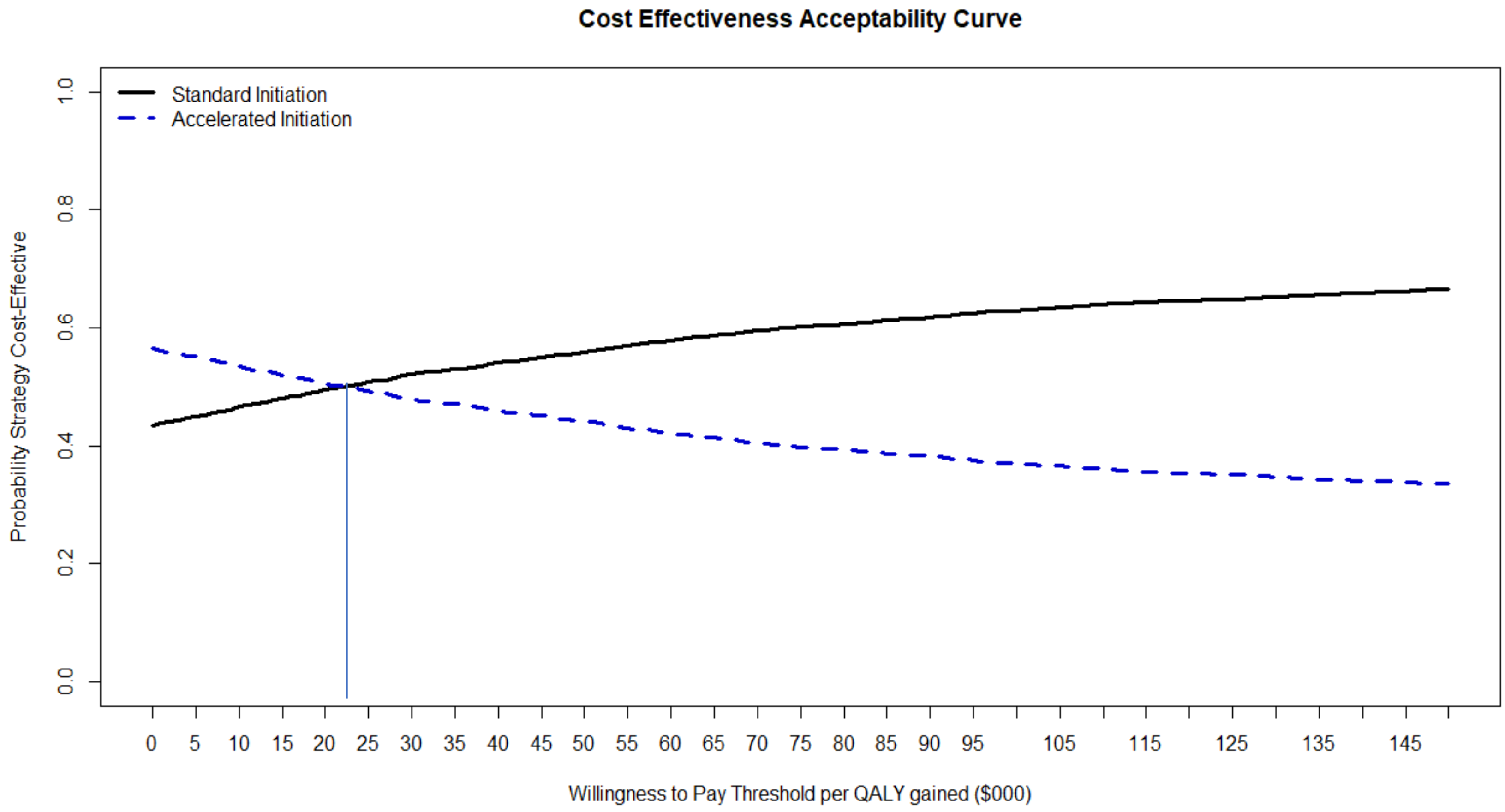

© 2025 Round J et al. *JAMA Network Open*.

eFigure 3: Cost-effectiveness acceptability curve, sensitivity analysis, varying monthly costs

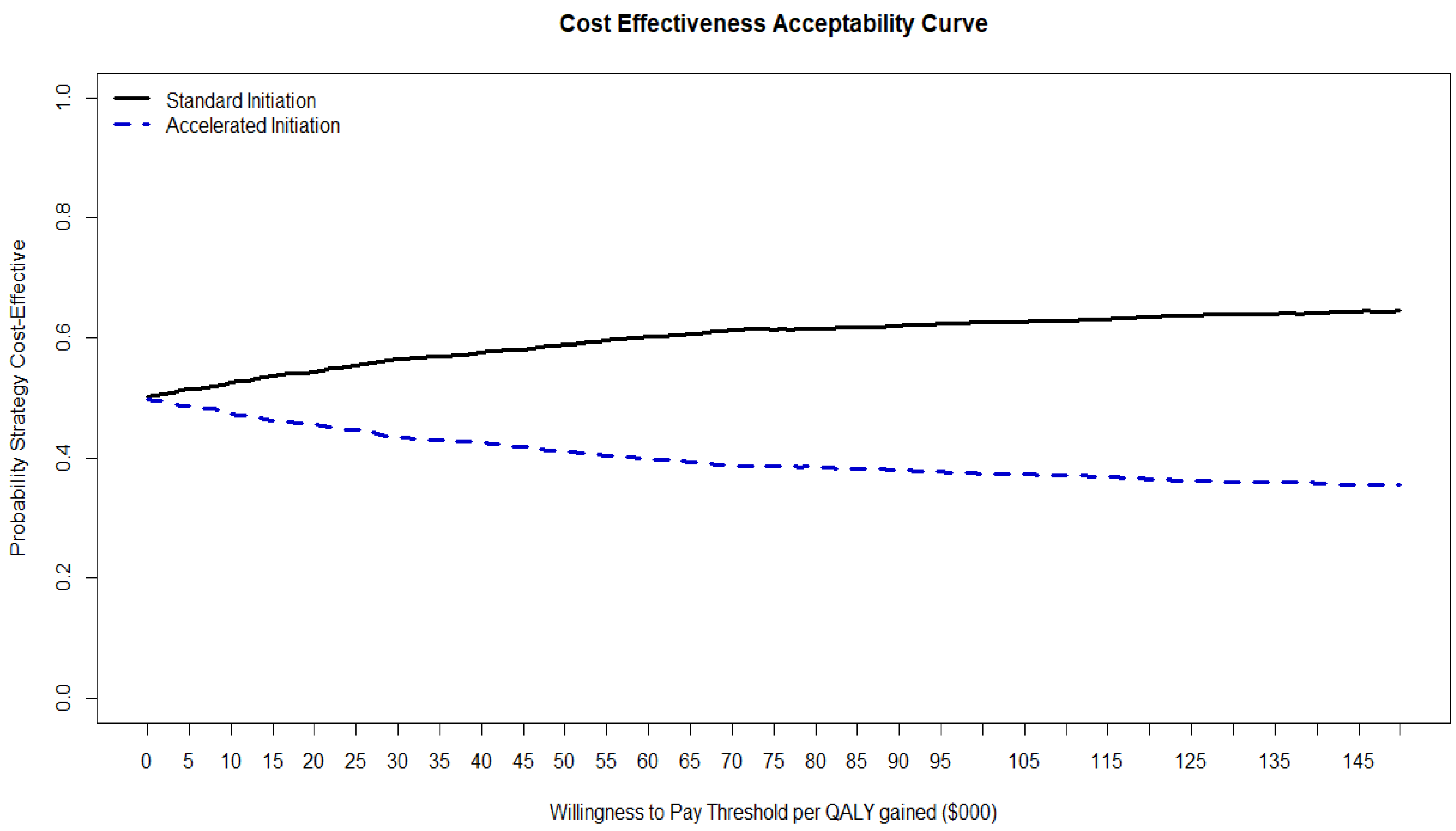

© 2025 Round J et al. *JAMA Network Open*.

eAppendix. Data elements

- The National Ambulatory Care Reporting System (NACRS) contains all ambulatory care utilization (emergency department visits, day procedures), including information on diagnoses, procedures and Comprehensive Ambulatory Classification System (CACS) grouper and resource intensity weight (RIW) [12].
- Discharge Abstract Database (DAD) contains all acute-care hospitalization details, including demographics, diagnoses, procedures, RIW, and Case Mix Group (CMG) classification [13].
- Practitioner Claims (PC) database contains fee-for-service claims information for physicians and other healthcare providers for government-insured health services [14].
- Pharmaceutical Information Network (PIN) contains prescribed medications dispensing details including dose, number dispensed and unit costs [15].
- Laboratory testing database, containing details of diagnostic and other laboratory tests [14].
- Population Registry and Vital Statistics records demographic and vital statistics for all inhabitants of Alb[25]erta [14].

© 2025 Round J et al. *JAMA Network Open*.

eTable 2: Ambulatory Care (NACRS)

Data Element Full Name	Description
Study ID	Study ID provided by requester.
Facility Site Identifier	Assigned by the submitter to uniquely identify the service location when different sites are defined under the same institution number (regional generic number).
Health Diagnosis Code 1 to 10	The diagnosis, condition, problem, or in some cases, the intervention, that is the reason for the services being provided to the recipient. The code is contained in the coding classification which is represented by the corresponding version number. Multiple diagnoses codes may be reported for one service episode.
Health Diagnosis Prefix Code 1 to 10	A character that provides additional information about a diagnosis code. Examples include "Q" for a query diagnosis or "C" for cause of death.
Health Status Triage Date	The date the recipient was triaged; applicable only to those recipients seen in an emergency department or a community urgent care centre.
Health Status Triage Level Code	The level of triage recorded for the recipient on this visit; applicable only to those recipients seen in an emergency department or a community urgent care centre. Triage is a method to prioritize patient care requirements and the triage level recorded here is in the form of the Canadian Acuity and Triage Scale tool (CTAS).
Health Status Triage Time	The time the recipient was triaged; applicable only to those recipients seen in an emergency department or a community urgent care centre. Time is reported using the 24 hour clock.
Location Facility Health Region Code Fiscal Year End	The code representing the health region of the reporting facility at fiscal year end.
Provider Role Code	Describes the role of the health care provider associated with the patient's care in any capacity.
Record Submission Facility Identifier	The submitting institution identifier in a format prescribed by CIHI comprised of Province number, Level of Care, and the provincially assigned unique facility number.
Record Submission Fiscal Year	Identifies the year the recipient was discharged from a facility according to the fiscal year (April 1 to March 31). For example, the fiscal year is recorded as 2003, for year 2002-2003.
Service Episode Ambulatory Canadian Institute for Health Information Group Code	CIHI assigned ambulatory care group derived from the reported primary MIS functional centre account code.
Service Episode Comprehensive Ambulatory Classification System Code	Developed by CIHI as part of a grouping methodology to create homogeneous patient clusters.
Service Episode Comprehensive Ambulatory Classification System Age Category Code	An age category code assigned as part of the CACS grouping methodology for ambulatory care.
Service Episode Comprehensive Ambulatory Classification System Intervention Canadian Classification of Interventions Code	The one CCI intervention code of those recorded that is used in Comprehensive Ambulatory Classification System (CACS) group assignment within the grouping methodology.
Service Episode Comprehensive Ambulatory Classification System Resource Intensity Weight Value	A weighting value assigned by CIHI to the record based on the grouping methodology. RIW values indicate expected relationships of costs between patient types. For instance, RIW 1.0000 = average expected cost per standard "average" ambulatory care case.
Service Episode Disposition Code	Identifies the service recipient's type of separation from the ambulatory care service.

© 2025 Round J et al. *JAMA Network Open*.

Data Element Full Name	Description
Service Episode End Date	The calendar date when the service recipient completed the current ambulatory care visit. If a date is not reported for an ambulatory care visit, the default of 9999/12/31 is recorded.
Service Episode End Time	The time the patient was formally discharged according to the 24-hour clock.
Service Episode Major Ambulatory Category Code	The MAC code is assigned as part of the ACCS grouping process. It is a broad level grouping classification consisting of similar ACCS group codes.
Service Episode Management Information System (MIS) Primary Code	Identifies the functional centre for which an ambulatory care service event is being reported, according to the Alberta MIS Chart of Accounts. It is intended to reflect the type of services provided.
Service Episode Mode of Service Code	Identifies the manner in which an ambulatory care service was provided to a service recipient such as face-to-face, group therapy or telephone etc.
Service Episode Record Grouper Identifier	The unique identifier for the grouper methodology.
Service Episode Start Date	The calendar date that the patient was officially registered as a recipient. If seen in emergency and then admitted, the admit date is the date the physician gives the order to admit. For newborns the admit date is the same as the date of birth.
Service Episode Start Time	The time when the service recipient was registered at the facility on the day the ambulatory care service was provided, recorded using the 24-hour clock.
Service Event Ambulance Type Code	Identifies whether or not a patient arrives via ambulance to the health care facility. Ambulance includes all licensed ambulances, inter-facility transfer service units and air ambulances having the capability of providing medical intervention to a service recipient en route to the destination.
Service Event Intervention Code 1 to 10	The operative or non-operative intervention(s) performed on the patient during his length of stay in the health care facility. The code is contained in the coding classification which is represented by the corresponding version number. There may be attribute or suffix codes which further define an intervention code. Multiple intervention codes may be reported for one service episode.
Emergency Indicator	Flag that indicates an Emergency Department visit
Total Cost	Total cost for selected records as provided by Alberta Health Services

© 2025 Round J et al. *JAMA Network Open*.

eTable 3: Inpatient (DADS)

Data Element Full Name	Description
Study ID	Study ID provided by requester.
Facility Institution Identifier Transfer From (MACAR)	Reported when a recipient is transferred from another health care facility or another level of care within the reporting facility for further treatment or hospitalization, but not if the transfer is from an ambulatory care or sub-acute area of the same facility.
Facility Institution Identifier Transfer To (MACAR)	Reported when a recipient is transferred to another health care facility or another level of care within the reporting facility for further treatment or hospitalization, but not if the transfer is to an ambulatory care or sub-acute area of the same facility.
Health Diagnosis Code 1 to 25	The diagnosis, condition, problem, or in some cases, the intervention, that is the reason for the services being provided to the recipient. The code is contained in the coding classification which is represented by the corresponding version number. Multiple diagnoses codes may be reported for one service episode.
Health Diagnosis Prefix Code 1 to 25	A character that provides additional information about a diagnosis code. Examples include "Q" for a query diagnosis or "C" for cause of death.
Health Diagnosis Type Code 1 to 25	An alpha or numeric code used to further describe the recorded diagnosis or condition. For example, the diagnosis type will differentiate the most responsible diagnosis from secondary diagnoses.
Record Submission Facility Identifier	The submitting institution identifier in a format prescribed by CIHI comprised of Province number, Level of Care, and the provincially assigned unique facility number.
Record Submission Fiscal Year	Identifies the year the recipient was discharged from a facility according to the fiscal year (April 1 to March 31). For example, the fiscal year is recorded as 2003, for year 2002-2003.
Service Episode Acute Length of Stay Days	Calculated by the Canadian Institute for Health Information (CIHI) to provide the total length of stay in days for the patient's hospitalization. The admission and discharge date fields are used.
Service Episode Admission Category Code	The patient classification on admission to a health care facility.
Service Episode Alternate Level of Care Length of Stay Days	The total number of days of a service episode reported with a patient service of 99 (Alternate Level of Care -ALC). Used to determine the number of days spent in an acute care bed while awaiting placement in an alternate level of care bed. ALC may be reported as the main patient service or as a service transfer.
Service Episode Case Mix Group Code	A CMG code is developed as part of a grouping methodology to create homogeneous patient clusters as per dates noted for each code. Inpatient records are reassigned group codes after correcting the transfer from/to institution numbers.
Service Episode Discharge Disposition Code	Identifies the location where the patient was discharged to or the status of the patient on discharge.
Service Episode End Date	The calendar date when the service recipient was formally discharged as an inpatient.
Service Episode End Time	The time the patient was formally discharged according to the 24-hour clock.
Service Episode Main Service Code	Identifies the focus of the treatment provided during a hospital stay; it is determined by the most responsible diagnosis. Examples of patient services include medicine, paediatrics, and obstetrics delivered.

© 2025 Round J et al. *JAMA Network Open*.

Data Element Full Name	Description
Service Episode Resource Intensity Level Code	A code that reflects the overall effect of all factors on the resource intensity weight of a particular case. It is a way of further distinguishing patients with higher resource use. For example, a Nonfactor case will have an RIL of 1. A case in a particular CMG and Age Group whose RIW has doubled due to the impact of the factors will have an RIL of 2.
Service Episode Resource Intensity Weight Atypical Code	A code that identifies the circumstance that caused the case to be considered atypical and therefore subject to a different calculation of the resource intensity weight for that case. This code represents Atypical cases which are cases that do not receive the normal or predicted course of treatment associated with inpatients in a specific CMG because they arrived at, or left, the facility in circumstances that made their total length of stay or costs unpredictable.
Service Episode Resource Intensity Weight Value	A weighting value assigned to the record based on the grouping methodology. Derived by Alberta or CIHI - the creator is specified in the Grouper table. Inpatient records are reassigned group codes after correcting the transfer from/to institution numbers. There is monontonicity in RIW value, that is the RIW for any given level of complexity with a CMG is never greater than the RIW at higher levels of complexity.
Service Episode Special Care Admit Date 1-3	The calendar date when the patient is admitted to a Special Care Unit (SCU).
Service Episode Special Care Admit Unit Code 1-3	Identifies the type of special care unit where the patient receives critical care.
Service Episode Special Care Discharge Date 1-3	The calendar date when the patient is discharged from or expires in the special care unit (SCU).
Service Episode Start Date	The calendar date that the patient was officially registered as a recipient. If seen in emergency and then admitted, the admit date is the date the physician gives the order to admit. For newborns, the admit date is the same as the date of birth.
Service Episode Start Time	The time when the service recipient was registered as an inpatient. For inpatients admitted subsequent to an emergency department visit, the start time is the time the physician gives the order to admit to acute care. For newborns, the start time is the same as the time of birth.
Service Episode Total Length of Stay Days	The total length of stay in days is a value calculated as the difference between the admit and discharge date. Invalid data will be recorded as -1.
Service Event Intervention Code 1 to 20	The operative or non-operative intervention(s) performed on the patient during his length of stay in the health care facility. The code is contained in the coding classification which is represented by the corresponding version number. There may be attribute or suffix codes which further define an intervention code. Multiple intervention codes may be reported for one service episode.
Service Event Intervention End Date 1 to 20	The date the patient left the operating room or other location following the operative episode of care. Used to standardize the capture of an operating room end date regardless of the number of interventions performed within that episode, to allow for accurate calculation of intervention episode duration.
Service Event Intervention End Time 1 to 20	The time the patient left the operating room or other location following the operative episode of care. Used to standardize the capture of an operating room end time regardless of the number of interventions performed within that episode, to allow for accurate calculation of intervention episode duration.
Service Event Intervention Start Date 1 to 20	The date the patient entered the operating room or other location for the operative episode of care. Used to standardize the capture of an operating room start date regardless of the number of interventions performed within that episode, to allow for accurate calculation of intervention episode duration.

Data Element Full Name	Description
Service Event Intervention Start Time 1 to 20	The time the patient entered the operating room or other location for the operative episode of care. Used to standardize the capture of an operating room start time regardless of the number of interventions performed within that episode, to allow for accurate calculation of intervention episode duration.
Total Cost	Total cost for selected records as provided by Alberta Health Services

© 2025 Round J et al. *JAMA Network Open*.

eTable 4: Long term care

Data Element Full Name	Description
Study ID	Study ID provided by requester.
Case Mix Index Data Value Number	Case Mix Index values assigned to the specific Resource Utilization Group (RUG) identifying the cost of services category.
Delivery Site Type	Site type assigned by the Delivery Site Registry (DSR) application.
Long Term Care Service Episode Admit Date	Date the resident was admitted to a long-term care facility. Identifies the start of a resident episode within ACCIS.
Long Term Care Service Episode Admit From Level of Care Code	Setting from which the resident came from prior to being admitted to the current long-term care facility.
Long Term Care Service Episode Discharge Date	Date on which a resident was permanently discharged from the facility. The date an individual is no longer considered a resident of the facility or the facility is no longer holding a bed for the resident.
Long Term Care Service Episode Discharge to Level of Care Code	Facility/level of care to which the resident is discharged or that the discharge is due to death.

© 2025 Round J et al. *JAMA Network Open*.

eTable 5: Pharmaceutical Information Network (PIN)

Data Element Full Name	Description
Study ID	Study ID provided by requester.
Component Sequence	A system generated sequence number assigned to each drug component in the compound. This value will always be 1 for dispenses that are associated to just one DIN (non-compounds).
Drug Identification Number	Identifies the Canadian Drug Identification number.
Dispensed Amount Quantity	The quantity of the dispensed product.
Dispensed Amount Unit Measure Code	Denotes the unit of measure that applies to the value in the Dispensed Quantity field. For drug products, the dosage form of the manufactured drug will determine the appropriate unit measure.
Dispense Date	The date on which the medication was dispensed.
Dispense Day Supply Quantity	The number of days that this dispensing event covers.
Dispense Day Supply Unit Measure Code	The unit in which the Day Supply Quantity is measured. All dispenses in PIN are implied to be in days.
Product Drug Key	Uniquely identifies the drug from the drug repository. This includes manufactured drug products, medical devices and compounds. This attribute can handle different length keys as determined by the Product Type Code.
Fiscal Year	The fiscal year of the dispense.

© 2025 Round J et al. *JAMA Network Open*.

eTable 6: Population registry—cumulative

Data Element Full Name	Description
Study ID	Study ID provided by requester.
Fiscal Year End Date	Fiscal year end date
Person Active Coverage Indicator Fiscal Year End	A flag that indicates whether or not a registrant is active at the end of the fiscal year.
Person Age at Fiscal Year End in Years	The age of the registrant (in years) determined at fiscal year end (March 31). Derived from the registrant's birth date.
Person Gender Code	A code depicting the biological sex of registrant.
Person Registration Eligibility and Premiums End Reason Code - Grouped	Grouped version of the code identifing cancellation of a family registration or deletion of dependant from the family registration. Derived from either the registration cancellation code or the deletion code.
Person Registration Eligibility and Premiums End Date	Date that a family registration coverage is terminated or a dependant is deleted from the family registration. Derived from either the registration cancellation date or the deletion date.
Alberta Health Services Continuum Zone Code	A level of the provincial health boundaries introduced in 2011, containing five entities. Zone Boundaries are based on aggregations of the Local Geography and SubZone boundaries. Derived.

© 2025 Round J et al. *JAMA Network Open*.

eTable 7: Population registry—monthly

Data Element Full Name	Description
Study ID	Study ID provided by requester.
Month End Date	Month end date
Person Age at Month End in Years	The age of the registrant (in years) determined at month end. Derived from the registrant's birth date.
Person Gender Code	A code depicting the biological sex of registrant.
Alberta Health Services Continuum Zone Code	A level of the provincial health boundaries introduced in 2011, containing five entities. Zone Boundaries are based on aggregations of the Local Geography and SubZone boundaries. Derived.

© 2025 Round J et al. *JAMA Network Open*.

eTable 8: Stakeholder registry

Data Element Full Name	Description
Study ID	Study ID provided by requester.
Death Date	Last End Date (REC_BUS_VER_END_DATE) where End Reason (REC_BUS_VER_END_RSN_CODE) = 'DECD'

© 2025 Round J et al. *JAMA Network Open*.

eTable 9: Practitioner claims (all)

Data Element Full Name	Description
Study ID	Study ID provided by requester.
Claims Assessment Event Final Amount	The final amount calculated after all variables that can affect the assessment amount have been considered; this includes override amounts entered by AH Claims Assessment staff. While the field is considered mandatory, the value can equal zero, as this is considered a valid amount.
Claims Assessment Event System Amount	The assessment amount (dollars) for the service as determined by the system. (No human intervention).
Delivery Site Facility Identifier (CLASS)	A number which uniquely identifies a registered physical facility where services are provided. This field will be populated with values of 0 for services provided in unregistered facilities (i.e. Home, Other). In these situations, the Delivery Site Unregistered Type will be populated.
Delivery Site Facility Type Code	A code which indicates the type of registered facility where a service was performed. It will only be populated when a Delivery Site Facility ID Number > 0 is present.
Delivery Site Functional Centre Code (CLASS)	A specific area within a facility where health services are provided. Each Functional Centre Code is of one Functional Centre Type. For example, Functional Centre Code EMRG (Emergency) is of Functional Centre Type AMBU (Ambulatory Care Services) only. The assigned Functional Centre Code may be considered by assessment rules during claims processing. This field will only be populated when the Facility Number is present.
Delivery Site Functional Centre Type Code	A code which characterizes a functional centre type in terms of the type of health services it provides. Each functional centre code is of one type. This field will be populated when Functional Centre Code is present.
Financial Resource Event Actual Paid Amount	The total amount that was paid for the claim.
Health Diagnosis ICD9 Code 1	Identifies the primary diagnosis on the claim as submitted by the Providers. There are three diagnostic code fields on a claim and these are considered to be primary, secondary and tertiary diagnoses; this field is considered to be the primary diagnosis.
Health Diagnosis ICD9 Code 2	Identifies the secondary diagnosis on the claim as submitted by the Providers.
Health Diagnosis ICD9 Code 3	Identifies the tertiary diagnosis on the claim as submitted by the Providers.
Health Service Category (CLASS)	A code which categorizes a health service (for example, a visit, a test, a minor procedure, or a major procedure). For major procedures, the code values indicate the applicable pre- and post-operative timeframe for which visits are included.
Health Service Canadian Classification of Procedures Extended Code	Identifies the service performed as defined in the Schedule of Benefits.
Health Service Provider Discipline Type (CLASS)	Indicates the type of discipline in which the practitioner provided the service. If a provider has more than one discipline, the system selects the discipline according to the skill of the provider and the service provided as indicated on the claim.
Program Alternate Payment Plan Indicator	Indicates the type of claim if billed under an APP Business Arrangement.
Program Subtype (CLASS)	Identifies the type of Alberta Health funding program to which the service provided belongs.
Provider Skill Type (CLASS)	Identifies the type of provider skill under which a service was performed.
Service Event End Date	The date on which the service event ended.

© 2025 Round J et al. *JAMA Network Open*.

Data Element Full Name	Description
Service Event Fiscal Year	The fiscal year in which the service occurred.

While most physician services in Canada are compensated on a FFS basis, some physicians are paid through alternative payment plans (APPs). Under APPs, the services provided by physicians are recorded as 'shadow billing'—claims submitted for tracking purposes without involving actual payment transactions. Consequently, cost estimates in the analysis assume that the average cost under APPs is equivalent to that under the FFS model.

© 2025 Round J et al. *JAMA Network Open*.

eTable 10: Laboratory

Data Element Full Name	Data Element Description
Recipient Anonymous Identifier	Scrambled Recipient Unique Lifetime Identifier
Collection Date	Date the specimen was collected.
Test Code	Code that identifies the ordered procedure.
Test Name	Name of the ordered procedure.
Test Result	The value reported for the resulted procedure.
Test Unit Of Measure	The unit of measure of the value reported for the resulted procedure.
Test Verify Date	The date the procedure was verified and/or performed.

© 2025 Round J et al. *JAMA Network Open*.
