# Supplementary material for: Cost-Utility Analysis of Accelerated and Standard Strategies for Renal Replacement Therapy Initiation
Source: JAMA Netw Open. 2025 Oct 3;8(10):e2535343. doi: 10.1001/jamanetworkopen.2025.35343 (PMC12495491; doi:10.1001/jamanetworkopen.2025.35343)
Supplement: Supplement 2. — STARRT-AKI Investigators [file jamanetwopen-e2535343-s002.pdf]

Supplemental Online Content: Nonauthor Collaborators
*First name, last name, and suffix (if applicable) are required and will appear in PubMed.

*Group Name(s): STARRT-AKI Investigators							
***First Name and Middle Initial(s)**	***Last Name**	***Suffix (eg, Jr, III)**	Academic Degrees	Institution	Location (city, state/province, country)	Role or Contribution, eg, chair, principal investigator	Group (if more than 1 Group listed in the byline) and/or Subgroup (eg, Steering Committee)
Rinaldo	Bellomo				Australia		Steering committee
Glenn M	Eastwood				Australia		
Leah	Peck				Australia		
Helen	Young				Australia		
Peter	Kruger				Australia		
Gordon	Laurie				Australia		
Emma	Saylor				Australia		
Jason	Meyer				Australia		
Ellen	Venz				Australia		
Krista	Wetzig				Australia		
Craig	French				Australia		
Forbes	McGain				Australia		
John	Mulder				Australia		
Gerard	Fennessy				Australia		
Sathyajith	Koottayi				Australia		
Samantha	Bates				Australia		
Miriam	Towns				Australia		
Rebecca	Morgan				Australia		
Anna	Tippett				Australia		
Andrew	Udy				Australia		
Chris	Mason				Australia		
Elisa	Licari				Australia		
Dashiell	Gantner				Australia		
Jason	McClure				Australia		
Alistair	Nichol				Australia		
Phoebe	McCracken				Australia		
Jasmin	Board				Australia		
Emma	Martin				Australia		
Shirley	Vallance				Australia		

Supplemental Online Content: Nonauthor Collaborators
*First name, last name, and suffix (if applicable) are required and will appear in PubMed.

*First Name and Middle Initial(s)	*Last Name	*Suffix (eg, Jr, III)	Academic Degrees	Institution	Location (city, state/province, country)	Role or Contribution, eg, chair, principal investigator	Group (if more than 1 Group listed in the byline) and/or Subgroup (eg, Steering Committee)
Meredith	Young				Australia		
Chelsey	Vladic				Australia		
Steve	McGloughlin				Australia		
David	Gattas				Australia		
Heidi	Buhr				Australia		
Jennifer	Coles				Australia		
Debra	Hutch				Australia		
James	Wun				Australia		
Louise	Cole				Australia		
Christina	Whitehead				Australia		
Julie	Lowrey				Australia		
Kristy	Masters				Australia		
Rebecca	Gresham				Australia		
Victoria	Campbell				Australia		
David	Gutierrez				Australia		
Jane	Brailsford				Australia		
Loretta	Forbes				Australia		
Lauren	Murray				Australia		
Teena	Maguire				Australia		
Martina	NiChonghaile				Australia		
Neil	Orford				Australia		
Allison	Bone				Australia		
Tania	Elderkin				Australia		
Tania	Salerno				Australia		
Tim	Chimunda				Australia		
Jason	Fletcher				Australia		
Emma	Broadfield				Australia		
Sanjay	Porwal				Australia		
Cameron	Knott				Australia		
Catherine	Boschert				Australia		

Supplemental Online Content: Nonauthor Collaborators
*First name, last name, and suffix (if applicable) are required and will appear in PubMed.

*First Name and Middle Initial(s)	*Last Name	*Suffix (eg, Jr, III)	Academic Degrees	Institution	Location (city, state/province, country)	Role or Contribution, eg, chair, principal investigator	Group (if more than 1 Group listed in the byline) and/or Subgroup (eg, Steering Committee)
Julie	Smith				Australia		
Angus	Richardson				Australia		
Dianne	Hill				Australia		
Graeme	Duke				Australia		
Peter	Oziemski				Australia		
Santiago	Cegarra				Australia		
Peter	Chan				Australia		
Deborah	Welsh				Australia		
Stephanie	Hunter				Australia		
Owen	Roodenburg				Australia		
John	Dyett				Australia		
Nicos	Kokotsis				Australia		
Max	Moser				Australia		
Yang	Yang				Australia		
Laven	Padayachee				Australia		
Joseph	Vetro				Australia		
Himangsu	Gangopadhyay				Australia		
Melissa	Kaufman				Australia		
Angaj	Ghosh				Australia		
Simone	Said				Australia		
Alpesh	Patel				Australia		
Shailesh	Bihari				Australia		
Elisha	Matheson				Australia		
Xia	Jin				Australia		
Tapaswi	Shrestha				Australia		
Kate	Schwartz				Australia		
Martin P	Gallagher				Australia		Steering committee
Rosalba	Cross				Australia		
Winston	Cheung				Australia		
Helen	Wong				Australia		

Supplemental Online Content: Nonauthor Collaborators
*First name, last name, and suffix (if applicable) are required and will appear in PubMed.

***First Name and Middle Initial(s)**	***Last Name**	***Suffix (eg, Jr, III)**	Academic Degrees	Institution	Location (city, state/province, country)	Role or Contribution, eg, chair, principal investigator	Group (if more than 1 Group listed in the byline) and/or Subgroup (eg, Steering Committee)
Mark	Kol				Australia		
Asim	Shah				Australia		
Amanda Y	Wang				Australia		
Zoltan	Endre				Australia		
Celia	Bradford				Australia		
Pierre	Janin				Australia		
Simon	Finfer				Australia		
Naomi	Diel				Australia		
Jonathan	Gatward				Australia		
Naomi	Hammond				Australia		
Anthony	Delaney				Australia		
Frances	Bass				Australia		
Elizabeth	Yarad				Australia		
Hergen	Buscher				Australia		
Claire	Reynolds				Australia		
Nerilee	Baker				Australia		
Michael	Joannidis				Austria		Steering committee
Romuald	Bellmann				Austria		
Andreas	Peer				Austria		
Julia	Hasslacher				Austria		
Paul	Koglberger				Austria		
Sebastian	Klein				Austria		
Klemens	Zotter				Austria		
Anna	Brandtner				Austria		
Armin	Finkenstedt				Austria		
Adelheid	Ditlbacher				Austria		
Frank	Hartig				Austria		
Dietmar	Fries				Austria		
Mirjam	Bachler				Austria		

Supplemental Online Content: Nonauthor Collaborators
*First name, last name, and suffix (if applicable) are required and will appear in PubMed.

***First Name and Middle Initial(s)**	***Last Name**	***Suffix (eg, Jr, III)**	Academic Degrees	Institution	Location (city, state/province, country)	Role or Contribution, eg, chair, principal investigator	Group (if more than 1 Group listed in the byline) and/or Subgroup (eg, Steering Committee)
Bettina	Schenk				Austria		
Martin	Wagner				Austria		
Philipp	Eller				Austria		
					Austria		
Thomas	Staudinger				Austria		
Esther	Tiller				Austria		
Peter	Schellongowski				Austria		
Andja	Bojic				Austria		
Eric A	Hoste				Belgium		Steering committee
Stephanie	Bracke				Belgium		
Luc	De Crop				Belgium		
Daisy	Vermeiren				Belgium		
Fernando	Thome				Brazil		
Bianca	Chiella				Brazil		
Lucia	Fendt				Brazil		
Veronica	Antunes				Brazil		
Jean-Philippe	Lafrance				Canada		
François	Lamontagne				Canada		Steering committee
Frédérick	D'Aragon				Canada		
Charles	St-Arnaud				Canada		
Michael	Mayette				Canada		
Élaine	Carbonnaeu				Canada		
Joannie	Marchand				Canada		
Marie-Hélène	Masse				Canada		
Marilène	Ladouceur				Canada		
Alexis F	Turgeon				Canada		
François	Lauzier				Canada		

Supplemental Online Content: Nonauthor Collaborators
*First name, last name, and suffix (if applicable) are required and will appear in PubMed.

***First Name and Middle Initial(s)**	***Last Name**	***Suffix (eg, Jr, III)**	Academic Degrees	Institution	Location (city, state/province, country)	Role or Contribution, eg, chair, principal investigator	Group (if more than 1 Group listed in the byline) and/or Subgroup (eg, Steering Committee)
David	Bellemare				Canada		
Charles L	Francouer				Canada		
Guillaume	LeBlanc				Canada		
Gabrielle	Guilbault				Canada		
Stéphanie	Grenier				Canada		
Eve	Cloutier				Canada		
Annick	Boivin				Canada		
Charles	Delisle-Thibault				Canada		
Panagiota	Giannakouros				Canada		
Olivier	Costerousse				Canada		
Jean-François	Cailhier				Canada		
François-Martin	Carrier				Canada		
Ali	Ghamraoui				Canada		
Martine	Lebrasseur				Canada		
Fatna	Benettaib				Canada		
Maya	Salamé				Canada		
Dounia	Boumahni				Canada		
Ying T	Sia				Canada		
Jean-François	Naud				Canada		
Isabelle	Roy				Canada		
Henry T	Stelfox				Canada		
Stacey	Ruddell				Canada		
Braden J	Manns				Canada		
Shelley	Duggan				Canada		
Dominic	Carney				Canada		
Jennifer	Barchard				Canada		
Richard P	Whitlock				Canada		
Emilie	Belley-Cote				Canada		
Nevena	Savija				Canada		
Alexandra	Sabev				Canada		

Supplemental Online Content: Nonauthor Collaborators
*First name, last name, and suffix (if applicable) are required and will appear in PubMed.

*First Name and Middle Initial(s)	*Last Name	*Suffix (eg, Jr, III)	Academic Degrees	Institution	Location (city, state/province, country)	Role or Contribution, eg, chair, principal investigator	Group (if more than 1 Group listed in the byline) and/or Subgroup (eg, Steering Committee)
Troy	Campbell				Canada		
Thais	Creary				Canada		
Kelson	Devereaux				Canada		
Shira	Brodutch				Canada		
Claudio	Rigatto				Canada		
Bojan	Paunovic				Canada		
Owen	Mooney				Canada		
Anna	Glybina				Canada		
Oksana	Harasemiw				Canada		
Michelle	Di Nella				Canada		
John	Harmon				Canada		
Navdeep	Mehta				Canada		
Louis	Lakatos				Canada		
Nicole	Haslam				Canada		
Francois	Lellouche				Canada		
Mathieu	Simon				Canada		
Ying	Tung				Canada		
Patricia	Lizotte				Canada		
Pierre-Alexandra	Bourchard				Canada		
Bram	Rochwerg				Canada		
Tim	Karachi				Canada		
Tina	Millen				Canada		
John	Muscedere				Canada		
David	Maslove				Canada		
J. Gordon	Boyd				Canada		
Stephanie	Sibley				Canada		
John	Drover				Canada		
Miranda	Hunt				Canada		
Ilinca	Georgescu				Canada		
Randy	Wax				Canada		

Supplemental Online Content: Nonauthor Collaborators
*First name, last name, and suffix (if applicable) are required and will appear in PubMed.

*First Name and Middle Initial(s)	*Last Name	*Suffix (eg, Jr, III)	Academic Degrees	Institution	Location (city, state/province, country)	Role or Contribution, eg, chair, principal investigator	Group (if more than 1 Group listed in the byline) and/or Subgroup (eg, Steering Committee)
Ilan	Lenga				Canada		
Kavita	Sridhar				Canada		
Andrew	Steele				Canada		
Kelly	Fusco				Canada		
Taneera	Ghate				Canada		
Michael	Tolibas				Canada		
Holly	Robinson				Canada		
Matthew A	Weir				Canada		Steering committee
Ravi	Taneja				Canada		
Ian M	Ball				Canada		
Amit	Garg				Canada		
Eileen	Campbell				Canada		
Athena	Ovsenek				Canada		
Sean M	Bagshaw				Canada	Co-Principal Investigator	Steering committee (co-chair)
Sean	van Diepen				Canada		
Nadia	Baig				Canada		
Sheldon	Magder				Canada		
Han	Yao				Canada		
Ahsan	Alam				Canada		
Josie	Campisi				Canada		
Erika	MacIntyre				Canada		
Ella	Rokosh				Canada		
Kimberly	Scherr				Canada		
Stephen	Lapinsky				Canada		
Sangeeta	Mehta				Canada		
Sumesh	Shah				Canada		
Daniel J	Niven				Canada		
Stacey	Ruddell				Canada		
Michael	Russell				Canada		

Supplemental Online Content: Nonauthor Collaborators
*First name, last name, and suffix (if applicable) are required and will appear in PubMed.

*First Name and Middle Initial(s)	*Last Name	*Suffix (eg, Jr, III)	Academic Degrees	Institution	Location (city, state/province, country)	Role or Contribution, eg, chair, principal investigator	Group (if more than 1 Group listed in the byline) and/or Subgroup (eg, Steering Committee)
Kym	Jim				Canada		
Gillian	Brown				Canada		
Kerry	Oxtoby				Canada		
Adam	Hall				Canada		
Luc	Benoit				Canada		
Colleen	Sokolowski				Canada		
Bhanu	Prasad				Canada		
Jag	Rao				Canada		
Shelley	Giebel				Canada		
Demetrio J	Kutsogiannis				Canada		
Patricia	Thompson				Canada		
Tayne	Thompson				Canada		
Robert	Cirone				Canada		
Kanthi	Kavikondala				Canada		
Mark	Soth				Canada		
France	Clarke				Canada		
Alyson	Takaoka				Canada		
Ron	Wald				Canada	Co-Principal Investigator	Steering committee (co-chair)
David	Mazer				Canada		
Karen	Burns				Canada		
Jan	Friedrich				Canada		
David	Klein				Canada		
Gyan	Sandhu				Canada		
Marlene	Santos				Canada		
Imrana	Khalid				Canada		
Jennifer	Hodder				Canada		
Peter	Dodek				Canada		
Najib	Ayas				Canada		
Victoria	Alcuaz				Canada		

Supplemental Online Content: Nonauthor Collaborators
*First name, last name, and suffix (if applicable) are required and will appear in PubMed.

***First Name and Middle Initial(s)**	***Last Name**	***Suffix (eg, Jr, III)**	Academic Degrees	Institution	Location (city, state/province, country)	Role or Contribution, eg, chair, principal investigator	Group (if more than 1 Group listed in the byline) and/or Subgroup (eg, Steering Committee)
Gabriel	Suen				Canada		
Oleksa	Rewa				Canada		
Gurmeet	Singh				Canada		
Sean	Norris				Canada		
Neil	Gibson				Canada		
Castro	Arias				Canada		
Aysha	Shami				Canada		
Celine	Pelletier				Canada		
Neill KJ	Adhikari				Canada		Steering committee
Alireza	Zahirieh				Canada		
Andre	Amaral				Canada		
Nicole	Marinoff				Canada		
Navjot	Kaur				Canada		
Adic	Perez				Canada		
Jane	Wang				Canada		
Gregory	Haljan				Canada		
Christopher	Condin				Canada		
Lauralyn	McIntyre				Canada		
Brigette	Gomes				Canada		
Rebecca	Porteous				Canada		
Irene	Watpool				Canada		
Swapnil	Hiremath				Canada		
Edward	Clark				Canada		
Margaret S	Herridge				Canada		
Felicity	Backhouse				Canada		
M. Elizabeth	Wilcox				Canada		
Karolina	Walczak				Canada		
Vincent	Ki				Canada		
Asheer	Sharman				Canada		
Martin	Romano				Canada		

Supplemental Online Content: Nonauthor Collaborators
*First name, last name, and suffix (if applicable) are required and will appear in PubMed.

*First Name and Middle Initial(s)	*Last Name	*Suffix (eg, Jr, III)	Academic Degrees	Institution	Location (city, state/province, country)	Role or Contribution, eg, chair, principal investigator	Group (if more than 1 Group listed in the byline) and/or Subgroup (eg, Steering Committee)
R.T. Noel	Gibney				Canada		
Adam S	Romanovsky				Canada		
Oleksa	Rewa				Canada		
Lorena	McCoshen				Canada		
Nadia	Baig				Canada		
Gordon	Wood				Canada		
Daniel	Ovakim				Canada		
Fiona	Auld				Canada		
Gayle	Carney				Canada		
Meili	Duan				China		
Xiaojun	Ji				China		
Dongchen	Guo				China		
Zhili	Qi				China		
Jin	Lin				China		
Meng	Zhang				China		
Lei	Dong				China		
Jingfeng	Liu				China		
Pei	Liu				China		
Deyuan	Zhi				China		
Guoqiang	Bai				China		
Yu	Qiu				China		
Ziqi	Yang				China		
Jing	Bai				China		
Zhuang	Liu				China		
Haizhou	Zhuang				China		
Haiman	Wang				China		
Jian	Li				China		
Mengya	Zhao				China		
Xiao	Zhou				China		

Supplemental Online Content: Nonauthor Collaborators
*First name, last name, and suffix (if applicable) are required and will appear in PubMed.

*First Name and Middle Initial(s)	*Last Name	*Suffix (eg, Jr, III)	Academic Degrees	Institution	Location (city, state/province, country)	Role or Contribution, eg, chair, principal investigator	Group (if more than 1 Group listed in the byline) and/or Subgroup (eg, Steering Committee)
Xianqing	Shi				China		
Baning	Ye				China		
Manli	Liu				China		
Jing	Wu				China		
Yongjian	Fu				China		
Dali	Long				China		
Yu	Pan				China		
Jinlong	Wang				China		
Huaxian	Mei				China		
Songsong	Zhang				China		
Mingxiang	Wen				China		
Enyu	Yang				China		
Sijie	Mu				China		
Jianquan	Li				China		
Tingting	Hu				China		
Bingyu	Qin				China		
Min	Li				China		
Cunzhen	Wang				China		
Xin	Dong				China		
Kaiwu	Wang				China		
Haibo	Wang				China		
Jianxu	Yang				China		
Bin	Du				China		Steering committee
Chuanyao	Wang				China		
Dongxin	Wang				China		
Nan	Li				China		
Zhui	Yu				China		
Song	Xu				China		
Lan	Yao				China		
Guo	Hou				China		

Supplemental Online Content: Nonauthor Collaborators
*First name, last name, and suffix (if applicable) are required and will appear in PubMed.

***First Name and Middle Initial(s)**	***Last Name**	***Suffix (eg, Jr, III)**	Academic Degrees	Institution	Location (city, state/province, country)	Role or Contribution, eg, chair, principal investigator	Group (if more than 1 Group listed in the byline) and/or Subgroup (eg, Steering Committee)
Zhou	Liu				China		
Liping	Lu				China		
Yingtao	Lian				China		
Chunting	Wang				China		
Jichen	Zhang				China		
Ruiqi	Ding				China		
Guoqing	Qi				China		
Qizhi	Wang				China		
Peng	Wang				China		
Zhaoli	Meng				China		
Man	Chen				China		
Xiaobo	Hu				China		
Xiandi	He				China		
Shibing	Zhao				China		
Lele	Hang				China		
Rui	Li				China		
Suhui	Qin				China		
Kun	Lu				China		
Shijuan	Dun				China		
Cheng	Liu				China		
Qi	Zhou				China		
Zhenzhen	Chen				China		
Jing	Mei				China		
Minwei	Zhang				China		
Hao	Xu				China		
Jincan	Lin				China		
Qindong	Shi				China		
Lijuan	Fu				China		
Qinjing	Zeng				China		
Hongye	Ma				China		

Supplemental Online Content: Nonauthor Collaborators
*First name, last name, and suffix (if applicable) are required and will appear in PubMed.

*First Name and Middle Initial(s)	*Last Name	*Suffix (eg, Jr, III)	Academic Degrees	Institution	Location (city, state/province, country)	Role or Contribution, eg, chair, principal investigator	Group (if more than 1 Group listed in the byline) and/or Subgroup (eg, Steering Committee)
Jinqi	Yan				China		
Lan	Gao				China		
Hongjuan	Liu				China		
Lei	Zhang				China		
Hao	Li				China		
Xiaona	He				China		
Jingqun	Fan				China		
Litao	Guo				China		
Yu	Liu				China		
Xue	Wang				China		
Jingjing	Sun				China		
Zhongmin	Liu				China		
Juan	Yang				China		
Lili	Ding				China		
Lulu	Sheng				China		
Xingang	Liu				China		
Jie	Yan				China		
Quihui	Wang				China		
Yifeng	Wang				China		
Dan	Zhao				China		
Shuangping	Zhao				China		
Chenghuan	Hu				China		
Jing	Li				China		
Fuxing	Deng				China		
Haibo	Qiu				China		Steering committee
Yi	Yang				China		
Min	Mo				China		
Chun	Pan				China		
Changde	Wu				China		
Yingzi	Huang				China		

Supplemental Online Content: Nonauthor Collaborators
*First name, last name, and suffix (if applicable) are required and will appear in PubMed.

*First Name and Middle Initial(s)	*Last Name	*Suffix (eg, Jr, III)	Academic Degrees	Institution	Location (city, state/province, country)	Role or Contribution, eg, chair, principal investigator	Group (if more than 1 Group listed in the byline) and/or Subgroup (eg, Steering Committee)
Lili	Huang				China		
Airan	Liu				China		
Ville	Pettilä				Finland		Steering committee
Suvi T	Vaara				Finland		Steering committee
Anna-Maija	Korhonen				Finland		
Sanna	Törnblom				Finland		
Sari	Sutinen				Finland		
Leena	Pettilä				Finland		
Jonna	Heinonen				Finland		
Eliria	Lappi				Finland		
Taria	Suhonen				Finland		
Sari	Karlsson				Finland		
Sanna	Hoppu				Finland		
Ville	Jalkanen				Finland		
Anne	Kuitunen				Finland		
Markus	Levoranta				Finland		
Jaakko	Långsjö				Finland		
Sanna	Ristimäki				Finland		
Kaisa	Malila				Finland		
Anna	Wootten				Finland		
Simo	Varila				Finland		
Mikko J	Järvisalo				Finland		
Outi	Inkinen				Finland		
Satu	Kentala				Finland		
Keijo	Leivo				Finland		
Paivi	Haltia				Finland		
Didier	Dreyfuss				France		Steering committee
Jean-Damien	Ricard				France		
Jonathan	Messika				France		

Supplemental Online Content: Nonauthor Collaborators
*First name, last name, and suffix (if applicable) are required and will appear in PubMed.

***First Name and Middle Initial(s)**	***Last Name**	***Suffix (eg, Jr, III)**	Academic Degrees	Institution	Location (city, state/province, country)	Role or Contribution, eg, chair, principal investigator	Group (if more than 1 Group listed in the byline) and/or Subgroup (eg, Steering Committee)
Abirami	Tiagarajah				France		
Malo	Emery				France		
Aline	Dechanet				France		
Coralie	Gernez				France		
Damien	Roux				France		
Laurent	Martin-Lefevre				France		
Maud	Fiancette				France		
Isabelle	Vinatier				France		
Jean Claude	Lacherade				France		
Gwenhaël	Colin				France		
Christine	Lebert				France		
Marie-Ange	Azais				France		
Aihem	Yehia				France		
Caroline	Pouplet				France		
Matthieu	Henry-Lagarrigue				France		
Amélie	Seguin				France		
Laura	Crosby				France		
Julien	Maizel				France		
Dimitri	Titeca-Beauport				France		
Alain	Combes				France		
Ania	Nieszkowska				France		
Paul	Masi				France		
Alexandre	Demoule				France		
Julien	Mayaux				France		
Martin	Dres				France		
Elise	Morawiec				France		
Maxens	Decalvele				France		
Suela	Demiri				France		
Morgane	Faure				France		
Clémence	Marios				France		

Supplemental Online Content: Nonauthor Collaborators
*First name, last name, and suffix (if applicable) are required and will appear in PubMed.

***First Name and Middle Initial(s)**	***Last Name**	***Suffix (eg, Jr, III)**	Academic Degrees	Institution	Location (city, state/province, country)	Role or Contribution, eg, chair, principal investigator	Group (if more than 1 Group listed in the byline) and/or Subgroup (eg, Steering Committee)
Maxime	Mallet				France		
Marie Amélie	Ordon				France		
Laura	Morizot				France		
Marie	Cantien				France		
François	Pousset				France		
Stéphane	Gaudry				France		Steering committee
Florent	Poirson				France		
Yves	Cohen				France		
Laurent	Argaud				France		
Martin	Cour				France		
Laurent	Bitker				France		
Marie	Simon				France		
Romain	Hernu				France		
Thomas	Baudry				France		
Sylvie	De La Salle				France		
Adrien	Robine				France		
Nicholas	Sedillot				France		
Xavier	Tchenio				France		
Camille	Bouisse				France		
Sylvie	Roux				France		
Saber Davide	Barbar				France		
Rémi	Trusson				France		
Fabienne	Tamion				France		
Steven	Grangé				France		
Dorothée	Carpentier				France		
Guillaume	Chevrel				France		
Luis	Ensenyat-Martin				France		
Sophie	Marque				France		
Jean-Pierre	Quenot				France		
Pascal	Andreu				France		

Supplemental Online Content: Nonauthor Collaborators
*First name, last name, and suffix (if applicable) are required and will appear in PubMed.

***First Name and Middle Initial(s)**	***Last Name**	***Suffix (eg, Jr, III)**	Academic Degrees	Institution	Location (city, state/province, country)	Role or Contribution, eg, chair, principal investigator	Group (if more than 1 Group listed in the byline) and/or Subgroup (eg, Steering Committee)
Auguste	Dargent				France		
Audrey	Large				France		
Nicolas	Chudeau				France		
Mickael	Landais				France		
Benoit	Derrien				France		
Jean Christophe	Callhan				France		
Christophe	Guitton				France		
Charlène	Le Moal				France		
Alain	Robert				France		
Karim	Asehnoune				France		
Raphaël	Cinotti				France		
Nicolas	Grillot				France		
Dominique	Demeure				France		
Christophe	Vinsonneau				France		
Imen	Rahmani				France		
Mehdi	Marzouk				France		
Thibault	Dekeyser				France		
Caroline	Sejourne				France		
Mélanie	Verlay				France		
Fabienne	Thevenin				France		
Lucie	Delecolle				France		
Didier	Thevenin				France		
Bertrand	Souweine				France		
Elisabeth	Coupez				France		
Mireille	Adda				France		
Jean-Pierre	Eraldi				France		
Antoine	Marchalot				France		
Nicolas	De Prost				France		
Armand Mekontso	Dessap				France		

Supplemental Online Content: Nonauthor Collaborators
*First name, last name, and suffix (if applicable) are required and will appear in PubMed.

***First Name and Middle Initial(s)**	***Last Name**	***Suffix (eg, Jr, III)**	Academic Degrees	Institution	Location (city, state/province, country)	Role or Contribution, eg, chair, principal investigator	Group (if more than 1 Group listed in the byline) and/or Subgroup (eg, Steering Committee)
Keyvan	Razazi				France		
Ferhat	Meziani				France		
Julie	Boisrame-Helms				France		
Raphael	Clere-Jehl				France		
Xavier	Delabranche				France		
Christine	Kummerlen				France		
Hamid	Merdji				France		
Alexandra	Monnier				France		
Yannick	Rabouel				France		
Hassene	Rahmani				France		
Hayat	Allam				France		
Samir	Chenaf				France		
Vincenta	Franja				France		
Bertrand	Pons				France		
Michel	Carles				France		
Frédéric	Martino				France		
Régine	Richard				France		
Benjamin	Zuber				France		
Guillaume	Lacave				France		
Karim	Lakhal				France		
Bertrand	Rozec				France		
Hoa	Dang Van				France		
Éric	Boulet				France		
Fouad	Fadel				France		
Cedric	Cleophax				France		
Nicolas	Dufour				France		
Caroline	Grant				France		
Marie	Thuong				France		
Jean	Reignier				France		
Emmanuel	Canet				France		

Supplemental Online Content: Nonauthor Collaborators
*First name, last name, and suffix (if applicable) are required and will appear in PubMed.

*First Name and Middle Initial(s)	*Last Name	*Suffix (eg, Jr, III)	Academic Degrees	Institution	Location (city, state/province, country)	Role or Contribution, eg, chair, principal investigator	Group (if more than 1 Group listed in the byline) and/or Subgroup (eg, Steering Committee)
Laurent	Nicolet				France		
Thierry	Boulain				France		
Mai-Anh	Nay				France		
Dalila	Benzekri				France		
François	Barbier				France		
Anne	Bretagnol				France		
Toufik	Kamel				France		
Armelle	Mathonnet				France		
Grégoire	Muller				France		
Marie	Skarzynski				France		
Julie	Rossi				France		
Amandine	Pradet				France		
Sandra	Dos Santos				France		
Aurore	Guery				France		
Lucie	Muller				France		
Luis	Felix				France		
Julien	Bohé				France		
Guillaume	Thiéry				France		
Nadia	Aissaoui				France		
Damien	Vimpere				France		
Morgane	Commeureuc				France		
Jean-Luc	Diehl				France		
Emmanuel	Guerot				France		
Orfeas	Liangos				Germany		
Monika	Wittig				Germany		
Alexander	Zarbock				Germany		
Mira	Küllmar				Germany		
Thomas	van Waegeningh				Germany		
Nadine	Rosenow				Germany		

Supplemental Online Content: Nonauthor Collaborators
*First name, last name, and suffix (if applicable) are required and will appear in PubMed.

*First Name and Middle Initial(s)	*Last Name	*Suffix (eg, Jr, III)	Academic Degrees	Institution	Location (city, state/province, country)	Role or Contribution, eg, chair, principal investigator	Group (if more than 1 Group listed in the byline) and/or Subgroup (eg, Steering Committee)
Alistair D	Nichol				Ireland		Steering committee
Kathy	Brickell				Ireland		
Peter	Doran				Ireland		
Patrick T	Murray				Ireland		
Giovanni	Landoni				Italy		
Rosalba	Lembo				Italy		
Alberto	Zangrillo				Italy		
Giacomo	Monti				Italy		
Margherita	Tozzi				Italy		
Matteo	Marzaroli				Italy		
Gaetano	Lombardi				Italy		
Gianluca	Paternoster				Italy		
Michelangelo	Vitiello				Italy		
Shay	McGuinness				New Zealand		
Rachael	Parke				New Zealand		
Magdalena	Butler				New Zealand		
Eileen	Gilder				New Zealand		
Keri-Anne	Cowdrey				New Zealand		
Samantha	Wallace				New Zealand		
Jane	Hallion				New Zealand		
Melissa	Woolett				New Zealand		
Philippa	Neal				New Zealand		
Karina	Duffy				New Zealand		
Stephanie	Long				New Zealand		
Colin	McArthur				New Zealand		
Catherine	Simmonds				New Zealand		
Yan	Chen				New Zealand		

Supplemental Online Content: Nonauthor Collaborators
*First name, last name, and suffix (if applicable) are required and will appear in PubMed.

*First Name and Middle Initial(s)	*Last Name	*Suffix (eg, Jr, III)	Academic Degrees	Institution	Location (city, state/province, country)	Role or Contribution, eg, chair, principal investigator	Group (if more than 1 Group listed in the byline) and/or Subgroup (eg, Steering Committee)
Rachael	McConnochie				New Zealand		
Lynette	Newby				New Zealand		
David	Knight				New Zealand		
Seton	Henderson				New Zealand		
Jan	Mehrtens				New Zealand		
Stacey	Morgan				New Zealand		
Anna	Morris				New Zealand		
Kymbalee	Vander Hayden				New Zealand		
Tara	Burke				New Zealand		
Matthew	Bailey				New Zealand		
Ross	Freebairn				New Zealand		
Lesley	Chadwick				New Zealand		
Penelope	Park				New Zealand		
Christine	Rolls				New Zealand		
Liz	Thomas				New Zealand		
Ulrike	Buehner				New Zealand		
Erin	Williams				New Zealand		
Jonathan	Albrett				New Zealand		
Simon	Kirkham				New Zealand		
Carolyn	Jackson				New Zealand		
Troy	Browne				New Zealand		
Jennifer	Goodson				New Zealand		
David	Jackson				New Zealand		
James	Houghton				New Zealand		
Owen	Callender				New Zealand		
Vicki	Higson				New Zealand		
Owen	Keet				New Zealand		
Clive	Dominy				New Zealand		
Paul	Young				New Zealand		
Anna	Hunt				New Zealand		

Supplemental Online Content: Nonauthor Collaborators
*First name, last name, and suffix (if applicable) are required and will appear in PubMed.

*First Name and Middle Initial(s)	*Last Name	*Suffix (eg, Jr, III)	Academic Degrees	Institution	Location (city, state/province, country)	Role or Contribution, eg, chair, principal investigator	Group (if more than 1 Group listed in the byline) and/or Subgroup (eg, Steering Committee)
Harriet	Judd				New Zealand		
Cassie	Lawrence				New Zealand		
Shaanti	Olatunji				New Zealand		
Yvonne	Robertson				New Zealand		
Charlotte	Latimer-Bell				New Zealand		
Deborah	Hendry				New Zealand		
Agnes	Mckay-Vucago				New Zealand		
Nina	Beehre				New Zealand		
Eden	Lesona				New Zealand		
Leanlove	Navarra				New Zealand		
Chelsea	Robinson				New Zealand		
Ryan	Jang				New Zealand		
Andrea	Junge				New Zealand		
Bridget	Lambert				New Zealand		
Antoine G	Schneider				Switzerland		Steering committee
Michel	Thibault				Switzerland		
Philippe	Eckert				Switzerland		
Sébastien	Kissling				Switzerland		
Erietta	Polychronopoulos				Switzerland		
Elettra	Poli				Switzerland		
Marco	Altarelli				Switzerland		
Madeleine	Schnorf				Switzerland		
Samia Abed	Mallaird				Switzerland		
Claudia	Heidegger				Switzerland		
Aurelie	Perret				Switzerland		
Philippe	Montillier				Switzerland		
Frederic	Sangla				Switzerland		
Seigenthaller	Neils				Switzerland		
Aude	De Watteville				Switzerland		

Supplemental Online Content: Nonauthor Collaborators
*First name, last name, and suffix (if applicable) are required and will appear in PubMed.

*First Name and Middle Initial(s)	*Last Name	*Suffix (eg, Jr, III)	Academic Degrees	Institution	Location (city, state/province, country)	Role or Contribution, eg, chair, principal investigator	Group (if more than 1 Group listed in the byline) and/or Subgroup (eg, Steering Committee)
Mandeep-Kaur	Phull				United Kingdom		
Aparna	George				United Kingdom		
Nauman	Hussain				United Kingdom		
Tatiana	Pogreban				United Kingdom		
Steve	Lobaz				United Kingdom		
Alison	Daniels				United Kingdom		
Mishell	Cunningham				United Kingdom		
Deborah	Kerr				United Kingdom		
Alice	Nicholson				United Kingdom		
Pradeep	Shanmugasundaram				United Kingdom		
Judith	Abrams				United Kingdom		
Katarina	Manso				United Kingdom		
Geraldine	Hambrook				United Kingdom		
Elizabeth	McKerrow				United Kingdom		
Juvy	Salva				United Kingdom		
Stephen	Foulkes				United Kingdom		
Matthew	Wise				United Kingdom		
Matt	Morgan				United Kingdom		
Jenny	Brooks				United Kingdom		
Jade	Cole				United Kingdom		
Tracy Michelle	Davies				United Kingdom		
Helen	Hill				United Kingdom		
Emma	Thomas				United Kingdom		
Marcela	Vizcaychipi				United Kingdom		
Behrad	Baharlo				United Kingdom		
Jaime	Carungcong				United Kingdom		
Patricia	Costa				United Kingdom		
Laura	Martins				United Kingdom		
Ritoo	Kapoor				United Kingdom		

Supplemental Online Content: Nonauthor Collaborators
*First name, last name, and suffix (if applicable) are required and will appear in PubMed.

*First Name and Middle Initial(s)	*Last Name	*Suffix (eg, Jr, III)	Academic Degrees	Institution	Location (city, state/province, country)	Role or Contribution, eg, chair, principal investigator	Group (if more than 1 Group listed in the byline) and/or Subgroup (eg, Steering Committee)
Tracy	Hazelton				United Kingdom		
Angela	Moon				United Kingdom		
Janine	Musselwhite				United Kingdom		
Ben	Shelley				United Kingdom		
Philip	McCall				United Kingdom		
Marlies	Ostermann				United Kingdom		Steering committee
Gill	Arbane				United Kingdom		
Aneta	Bociek				United Kingdom		
Martina	Marotti				United Kingdom		
Rosario	Lim				United Kingdom		
Sara	Campos				United Kingdom		
Neus Grau	Novellas				United Kingdom		
Armando	Cennamo				United Kingdom		
Andrew	Slack				United Kingdom		
Duncan	Wyncoll				United Kingdom		
Luigi	Camporota				United Kingdom		
Simon	Sparkes				United Kingdom		
Rosalinde	Tilley				United Kingdom		
Austin	Rattray				United Kingdom		
Gayle	Moreland				United Kingdom		
Jane	Duffy				United Kingdom		
Elizabeth	McGonigal				United Kingdom		
Philip	Hopkins				United Kingdom		
Clare	Finney				United Kingdom		
John	Smith				United Kingdom		
Harriet	Noble				United Kingdom		
Hayley	Watson				United Kingdom		
Claire-Louise	Harris				United Kingdom		
Emma	Clarey				United Kingdom		
Eleanor	Corcoran				United Kingdom		

Supplemental Online Content: Nonauthor Collaborators
*First name, last name, and suffix (if applicable) are required and will appear in PubMed.

*First Name and Middle Initial(s)	*Last Name	*Suffix (eg, Jr, III)	Academic Degrees	Institution	Location (city, state/province, country)	Role or Contribution, eg, chair, principal investigator	Group (if more than 1 Group listed in the byline) and/or Subgroup (eg, Steering Committee)
James	Beck				United Kingdom		
Clare	Howcroft				United Kingdom		
Nora	Youngs				United Kingdom		
Elizabeth	Wilby				United Kingdom		
Bethan	Ogg				United Kingdom		
Adam	Wolverson				United Kingdom		
Sandra	Lee				United Kingdom		
Susie	Butler				United Kingdom		
Maryanne	Okubanjo				United Kingdom		
Julia	Hindle				United Kingdom		
Ingeborg	Welters				United Kingdom		
Karen	Williams				United Kingdom		
Emily	Johnson				United Kingdom		
Julie	Patrick-Heselton				United Kingdom		
David	Shaw				United Kingdom		
Victoria	Waugh				United Kingdom		
Richard	Stewart				United Kingdom		
Esther	Mwaura				United Kingdom		
Lynn	Wren				United Kingdom		
Louise	Mew				United Kingdom		
Sara-Beth	Sutherland				United Kingdom		
Jane	Adderley				United Kingdom		
Jim	Ruddy				United Kingdom		
Margaret	Harkins				United Kingdom		
Callum	Kaye				United Kingdom		
Teresa	Scott				United Kingdom		
Wendy	Mitchell				United Kingdom		
Felicity	Anderson				United Kingdom		
					United Kingdom		
Fiona	Willox				United Kingdom		

Supplemental Online Content: Nonauthor Collaborators
*First name, last name, and suffix (if applicable) are required and will appear in PubMed.

*First Name and Middle Initial(s)	*Last Name	*Suffix (eg, Jr, III)	Academic Degrees	Institution	Location (city, state/province, country)	Role or Contribution, eg, chair, principal investigator	Group (if more than 1 Group listed in the byline) and/or Subgroup (eg, Steering Committee)
Vijay	Jagannathan				United Kingdom		
Michele	Clark				United Kingdom		
Sarah	Purv				United Kingdom		
Andrew	Sharman				United Kingdom		
Megan	Meredith				United Kingdom		
Lucy	Ryan				United Kingdom		
Louise	Conner				United Kingdom		
Cecilia	Peters				United Kingdom		
Dan	Harvey				United Kingdom		
Ashraf	Roshdy				United Kingdom		
Amy	Collins				United Kingdom		
Malcolm	Sim				United Kingdom		
Steven	Henderson				United Kingdom		
Nigel	Chee				United Kingdom		
Sally	Pitts				United Kingdom		
Katie	Bowman				United Kingdom		
Maria	Dilawershah				United Kingdom		
Luke	Vamplew				United Kingdom		
Elizabeth	Howe				United Kingdom		
Paula	Rogers				United Kingdom		
Clara	Hernandez				United Kingdom		
Clara	Prendergast				United Kingdom		
Jane	Benton				United Kingdom		
Alex	Rosenberg				United Kingdom		
Lui G	Forni				United Kingdom		
Alice	Grant				United Kingdom		
Paula	Carvelli				United Kingdom		
Ajay	Raithatha				United Kingdom		
Sarah	Bird				United Kingdom		
Max	Richardson				United Kingdom		

Supplemental Online Content: Nonauthor Collaborators
*First name, last name, and suffix (if applicable) are required and will appear in PubMed.

***First Name and Middle Initial(s)**	***Last Name**	***Suffix (eg, Jr, III)**	Academic Degrees	Institution	Location (city, state/province, country)	Role or Contribution, eg, chair, principal investigator	Group (if more than 1 Group listed in the byline) and/or Subgroup (eg, Steering Committee)
Matthew	Needham				United Kingdom		
Claire	Hirst				United Kingdom		
Jonathan	Ball				United Kingdom		
Susannah	Leaver				United Kingdom		
Luisa	Howlett				United Kingdom		
Carlos	Castro Delgado				United Kingdom		
Sarah	Farnell-Ward				United Kingdom		
Helen	Farrah				United Kingdom		
Geraldine	Gray				United Kingdom		
					United Kingdom		
Gipsy	Joseph				United Kingdom		
Francesca	Robinson				United Kingdom		
Ascanio	Tridente				United Kingdom		
Clare	Harrop				United Kingdom		
Karen	Shuker				United Kingdom		
Derek	McLaughlan				United Kingdom		
Judith	Ramsey				United Kingdom		
Sharon	Meehan				United Kingdom		
Bernd Oliver	Rose				United Kingdom		
Rosie	Reece-Anthony				United Kingdom		
Babita	Gurung				United Kingdom		
Tony	Whitehouse				United Kingdom		
Catherine	Snelson				United Kingdom		
Tonny	Veenith				United Kingdom		
Andy	Johnston				United Kingdom		
Lauren	Cooper				United Kingdom		
Ron	Carrera				United Kingdom		
Karen	Ellis				United Kingdom		
Emma	Fellows				United Kingdom		
Samanth	Harkett				United Kingdom		

Supplemental Online Content: Nonauthor Collaborators
*First name, last name, and suffix (if applicable) are required and will appear in PubMed.

*First Name and Middle Initial(s)	*Last Name	*Suffix (eg, Jr, III)	Academic Degrees	Institution	Location (city, state/province, country)	Role or Contribution, eg, chair, principal investigator	Group (if more than 1 Group listed in the byline) and/or Subgroup (eg, Steering Committee)
Colin	Bergin				United Kingdom		
Elaine	Spruce				United Kingdom		
Liesl	Despy				United Kingdom		
Stephanie	Goundry				United Kingdom		
Natalie	Dooley				United Kingdom		
Tracy	Mason				United Kingdom		
Amy	Clark				United Kingdom		
Gemma	Dignam				United Kingdom		
Geraldine	Ward				United Kingdom		
Ben	Attwood				United Kingdom		
Penny	Parsons				United Kingdom		
Sophie	Mason				United Kingdom		
Michael	Margarson				United Kingdom		
Jenny	Lord				United Kingdom		
Philip	McGlone				United Kingdom		
Luke E	Hodgson				United Kingdom		
Indra	Chadbourn				United Kingdom		
Raquel	Gomez				United Kingdom		
Jordi	Margalef				United Kingdom		
Rinus	Pretorius				United Kingdom		
Alexandra	Hamshere				United Kingdom		
Joseph	Carter				United Kingdom		
Hazel	Cahill				United Kingdom		
Lia	Grainger				United Kingdom		
Kate	Howard				United Kingdom		
Greg	Forshaw				United Kingdom		
Zoe	Guy				United Kingdom		
					United Kingdom		
Kianoush B	Kashani				United States		
Robert C	Albright				United States		

Supplemental Online Content: Nonauthor Collaborators
*First name, last name, and suffix (if applicable) are required and will appear in PubMed.

***First Name and Middle Initial(s)**	***Last Name**	***Suffix (eg, Jr, III)**	Academic Degrees	Institution	Location (city, state/province, country)	Role or Contribution, eg, chair, principal investigator	Group (if more than 1 Group listed in the byline) and/or Subgroup (eg, Steering Committee)
Amy	Amsbaugh				United States		
Anita	Stoltenberg				United States		
Alexander S	Niven				United States		
Matthew	Lynch				United States		
AnnMarie	O'Mara				United States		
Syed	Naeem				United States		
Sairah	Sharif				United States		
Joyce	McKenney Goulart				United States		
Matthew	Lynch				United States		
AnnMarie	O'Mara				United States		
Syed	Naeem				United States		
Sairah	Sharif				United States		
Ashita	Tolwani				United States		
Claretha	Lyas				United States		
Laura	Latta				United States		
Azra	Bihorac				United States		
Haleh	Hashemighouchani				United States		
Philip	Efron				United States		
Matthew	Ruppert				United States		
Julie	Cupka				United States		
Sean	Kiley				United States		
Joshua	Carson				United States		
Peggy	White				United States		
George	Omalay				United States		
Sherry	Brown				United States		
Laura	Velez				United States		
Alina	Marceron				United States		
Javier A	Neyra				United States		
Juan Carlos	Aycinena				United States		
Madona	Elias				United States		

Supplemental Online Content: Nonauthor Collaborators
*First name, last name, and suffix (if applicable) are required and will appear in PubMed.

***First Name and Middle Initial(s)**	***Last Name**	***Suffix (eg, Jr, III)**	Academic Degrees	Institution	Location (city, state/province, country)	Role or Contribution, eg, chair, principal investigator	Group (if more than 1 Group listed in the byline) and/or Subgroup (eg, Steering Committee)
Victor M	Ortiz-Soriano				United States		
Caroline	Hauschild				United States		
Robert	Dorfman				United States		
Kathleen D	Liu						Steering committee
Daniel F	McAuley						Steering committee
Shay P	McGuinness						Steering committee
Orla M	Smith						Steering committee
