# Supplementary material for: Cost-Utility Analysis of Accelerated and Standard Strategies for Renal Replacement Therapy Initiation
Source: JAMA Netw Open. 2025 Oct 3;8(10):e2535343. doi: 10.1001/jamanetworkopen.2025.35343 (PMC12495491; doi:10.1001/jamanetworkopen.2025.35343)
Supplement: Supplement 3. — Data Sharing Statement [file jamanetwopen-e2535343-s003.pdf]

Data Sharing Statement

Round. Cost-Utility Analysis of Accelerated and Standard Strategies for Renal Replacement Therapy Initiation. *JAMA Netw Open*. Published October 03, 2025.
doi:10.1001/jamanetworkopen.2025.35343

Data
Data available: No

Additional Information
Explanation for why data not available: The terms of the agreement under which we received the data from the Government of Alberta do not permit sharing.
